# Comparative Analysis of Behavioral Models for Adaptive Learning in Changing Environments

**DOI:** 10.3389/fncom.2016.00033

**Published:** 2016-04-20

**Authors:** Dimitrije Marković, Stefan J. Kiebel

**Affiliations:** Department of Psychology, Technische Universität Dresden Dresden, Germany

**Keywords:** decision making, changing environments, change point models, Hierarchical Gaussian Filters, Bayesian inference, maximum-likelihood estimate, Bayesian model comparison

## Abstract

Probabilistic models of decision making under various forms of uncertainty have been applied in recent years to numerous behavioral and model-based fMRI studies. These studies were highly successful in enabling a better understanding of behavior and delineating the functional properties of brain areas involved in decision making under uncertainty. However, as different studies considered different models of decision making under uncertainty, it is unclear which of these computational models provides the best account of the observed behavioral and neuroimaging data. This is an important issue, as not performing model comparison may tempt researchers to over-interpret results based on a single model. Here we describe how in practice one can compare different behavioral models and test the accuracy of model comparison and parameter estimation of Bayesian and maximum-likelihood based methods. We focus our analysis on two well-established hierarchical probabilistic models that aim at capturing the evolution of beliefs in changing environments: Hierarchical Gaussian Filters and Change Point Models. To our knowledge, these two, well-established models have never been compared on the same data. We demonstrate, using simulated behavioral experiments, that one can accurately disambiguate between these two models, and accurately infer free model parameters and hidden belief trajectories (e.g., posterior expectations, posterior uncertainties, and prediction errors) even when using noisy and highly correlated behavioral measurements. Importantly, we found several advantages of Bayesian inference and Bayesian model comparison compared to often-used Maximum-Likelihood schemes combined with the Bayesian Information Criterion. These results stress the relevance of Bayesian data analysis for model-based neuroimaging studies that investigate human decision making under uncertainty.

## 1. Introduction

To efficiently operate and survive in our everyday environment it is essential to quickly adapt to any unexpected changes that might occur. Numerous studies investigated computational principles and neuronal mechanism that underlie human decision making in changing environments (Angela, 2007; Behrens et al., 2007; Doya, 2008; Summerfield et al., 2011; Payzan-LeNestour et al., 2013). The large interest in this topic has led to the development of several behavioral models that can elucidate various features of human decision making under uncertainty (Yu and Dayan, 2005; Nassar et al., 2010; Payzan-LeNestour, 2010; Mathys et al., 2011; Payzan-LeNestour and Bossaerts, 2011; Wilson and Niv, 2011; Wilson et al., 2013; Glaze et al., 2015). The main premise of these models is the adaptive representation of the state of the world that can compensate for various sources of uncertainty in the environment (Bland and Schaefer, 2012). In recent years several of these models have been applied to model-based behavioral and neuroimaging data analysis, which provided valuable insights into the underlying computational processes and their neural mechanisms (Behrens et al., 2007; Summerfield et al., 2011; Nassar et al., 2012; Iglesias et al., 2013; Payzan-LeNestour et al., 2013; McGuire et al., 2014; Vossel et al., 2014).

However, in a typical study, the authors focused on one specific model that describes the evolution of beliefs and did not consider alternative formulations that may equally well describe decision making under uncertainty. The lack of model comparisons in this field casts some doubt on whether one can draw specific conclusions from the data, as there might be an alternative, well-established model that explains the data even better. An interesting question is whether there are specific components in each model that are not predicted by other models and which can be used for disambiguating among models. Critically, such model comparisons may tell us whether the data allow for the identification of a particular model and its computational mechanism. Importantly, for model comparison the inference methods used should be accurate. Otherwise a model comparison may not be sensible, as any difference between models may also be caused by inaccurately inferred parameters. Here we will investigate whether such accurate model selection is possible when considering different models of decision making under uncertainty.

To avoid making the analysis overly complex we focused on two well-established models: (i) the Hierarchical Gaussian Filters (HGF) (Mathys et al., 2011, 2014), and (ii) Change Point Models (CPM) (Nassar et al., 2010). To our knowledge these are the only two models that were applied multiple times in behavioral and neuroimaging studies (Nassar et al., 2012; Iglesias et al., 2013; Diaconescu et al., 2014; McGuire et al., 2014; Paliwal et al., 2014; Vossel et al., 2014, 2015) that investigated decision making in changing environments. Although these models are well-established, to the best of our knowledge and surprisingly, they have never been compared on the same behavioral or neuroimaging data. Both models assume a hierarchical, generative model of percepts, where different hierarchical levels account for expected and unexpected changes in the environment. Model inversion of these hierarchical generative models results in delta-like learning rules with adaptive learning rates. These learning rules define perceptual models, that is, mapping of sensory stimuli to beliefs about the current state of the world (Nassar et al., 2010; Mathys et al., 2011).

Importantly, we have re-formulated the CPM within the framework of Bayesian variational inference that was also used for deriving the HGF. This formulation of both models within the same inference framework allowed us a direct comparison of free model parameters and perceptual variables (e.g., posterior expectation and uncertainty, and learning rates) between different models and at different levels of the hierarchy. Although we perform a comparative analysis on just two behavioral models, the analysis presented here can be extended to an arbitrary large number of behavioral models, as long as all the models in the set can be formulated as a sequential decision making process with probabilistically generated responses.

Using synthetic data, we have tested two different methods for model inference and model comparison: (i) Maximum likelihood estimate (MLE), which is used quite often in model-based data analysis (Behrens et al., 2007; Summerfield et al., 2011; Wilson et al., 2013) in spite of being known as inaccurate when applied to fitting dynamical systems to noisy data (Horbelt, 2001; Judd, 2007); and (ii) Bayesian inference (BI), based on a recently proposed meta-Bayesian framework, the so-called “Observing the Observer” framework (Daunizeau et al., 2010a,b), which is known to provide high accuracy for parameter estimation and model comparison even for a large number of free model parameters (Iglesias et al., 2013; Mathys et al., 2014; Vossel et al., 2014).

In what follows we will demonstrate that, using a synthetic experiment and simulated behavioral data, it is possible to accurately infer free parameters of the two behavioral models, and disambiguate between models (when constraining experimental conditions to a set of values typically used in behavioral experiments). Importantly, unlike the MLE method, the BI method can accurately perform model comparison and parameter estimation, independent of the duration of the experiment (number of behavioral responses), or the amount of noise (deviations from the optimal response) in the responses of the simulated participants. Given these results, we conclude that Bayesian inference and model comparison is essential for analyzing and designing decision making experiments under uncertainty.

## 2. Results

**Figure 1:**
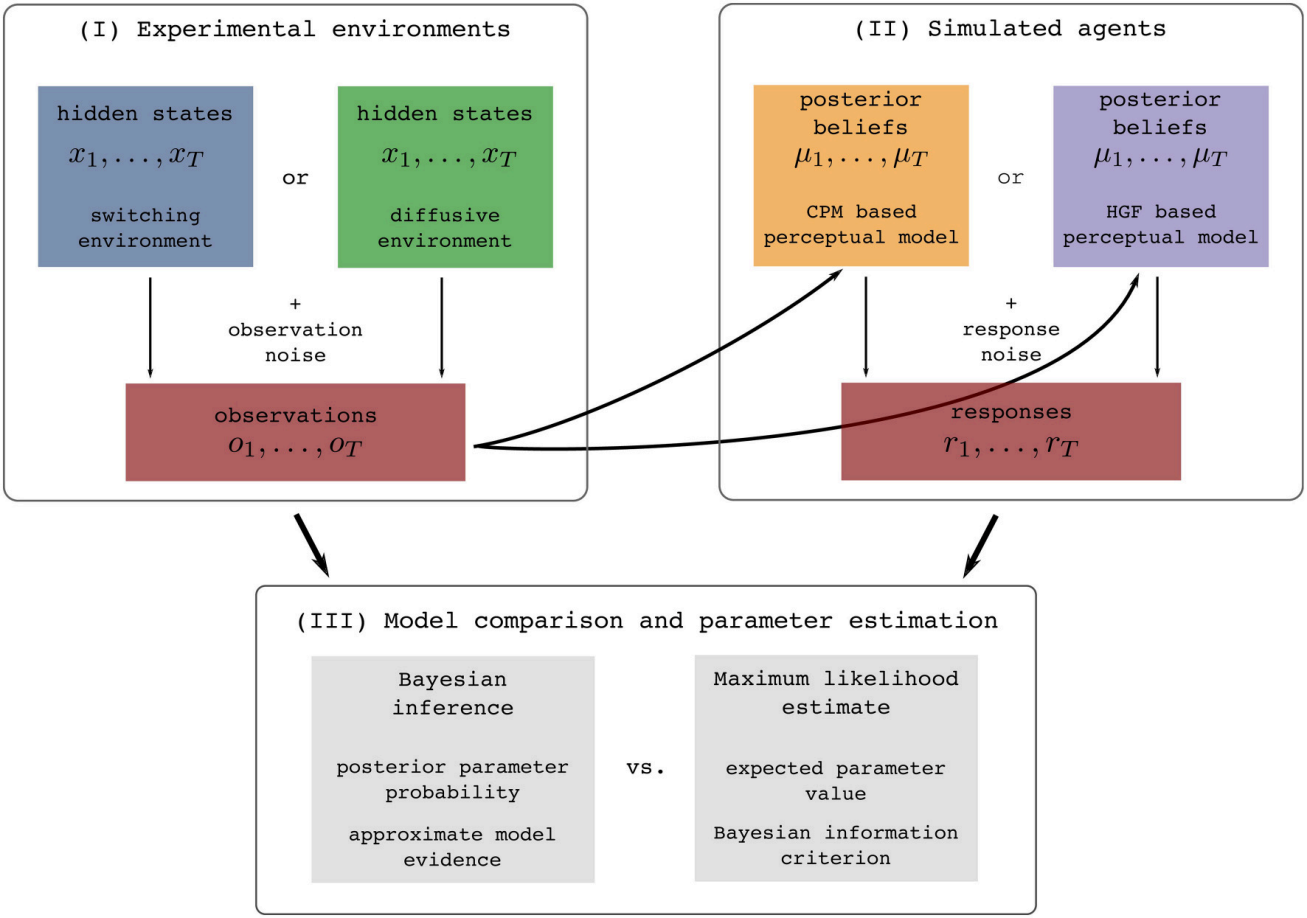
**Illustration of the experimental procedure: (I) Simulation of the two experimental environments (see Section 2.2 for details), (II) simulation of behavioral responses of simulated agents from one of the two behavioral models given the generated sensory stimuli (see Section 2.3 for details), and (III) the subsequent identification of the model that generated behavior using two different methodologies (see Section 4.2 for details)**. The colors assigned to different experimental environments (blue and green) or behavioral models (yellow and violet) will be used throughout the paper when presenting the results that are either conditioned on the experimental environment or on the behavioral models.

In this section we will first briefly describe the two so-called perceptual models, a response model, and the types of noisy environments in which we generated sensory stimuli. To clarify this procedure for the reader we show in Figure 1 an illustration of the full process of generating stimuli, simulating behavioral responses, and analyzing the generated data.

There are three steps of how we perform the analysis (Figure 1-I). The first step of the experimental procedure consists of generating sensory stimuli (observations) *o*_*t*_, i.e., we simulate the experimental environment. The quantity *o*_*t*_ can in practice be related to an arbitrary feature of the real world stimuli [e.g., a position of a dot on a screen (Nassar et al., 2010, 2012; Wilson et al., 2013), a location cue (Vossel et al., 2014) or an amount of reward (Iglesias et al., 2013)]. In the second step, we simulate the behavior of agents, e.g., a participant. To do this, we first present the generated sequence of sensory stimuli *O*_*T*_ to each of the two perceptual models (simulated agents), where by perceptual model we denote a specific mapping from observations to posterior beliefs. This mapping is defined either using the HGF or CPM model. Using the simulated belief process (that simulates how a participant may represent the presented sequence of stimuli), we generate behavioral responses *r*_*t*_ (Figure 1-II). The behavioral responses are obtained using a response model that maps posterior beliefs into responses. In the third step, given the generated set of sensory stimuli and behavioral responses we infer the corresponding parameters and trajectories of the internal variables of the two models (Figure 1-III). In this final step we make an inference about the hidden inference process, i.e., what model the simulated participant used (either HGF or CPM), and which parameter values best explain the data. We will now describe in more detail all components of this analysis.

### 2.1. Brief introduction of perceptual models

HGF are based on the assumption that percepts in a dynamic environment can be modeled as a hierarchy of coupled Gaussian random walks (Behrens et al., 2007; Mathys et al., 2011, 2014). This hierarchical representation can account for various forms of uncertainty that can influence perception and decision making (Yu and Dayan, 2005; Bland and Schaefer, 2012): The higher levels of the hierarchy encode the volatility of the corresponding state of the world and the speed at which the volatility changes. The higher is the volatility, the bigger is the expected change in the corresponding state of the world.

Although the hierarchy can in principle be extended to any number of levels (Mathys et al., 2014), we will consider here the simple case of a two-level HGF implementation, which is typically used in experimental studies (e.g., Iglesias et al., 2013; Vossel et al., 2014). The generative model is defined as

(1) $$\begin{array}{l} x_{t}^{\left(2\right)}=x_{t-1}^{\left(2\right)}+\sqrt{\eta }\cdot n_{t}^{\left(2\right)},\\ x_{t}^{\left(1\right)}=x_{t-1}^{\left(1\right)}+e^{\frac{x_{t}^{\left(2\right)}}{2}}\cdot n_{t}^{\left(1\right)},\\ \;o_{t}=x_{t}^{\left(1\right)}+\sqrt{s}\cdot n_{t}^{\left(o\right)}. \end{array}$$

Here *o* denotes observations, subscript *t* denotes discrete time points (e.g., experimental trials), *x*^(1)^ and *x*^(2)^ the hidden states at different levels of hierarchy (*x*^(1)^ is associated with the hidden state of the world and *x*^(2)^ with the volatility of the hidden state); $n_{t}^{\left(o\right)}$, $n_{t}^{\left(1\right)}$, and $n_{t}^{\left(2\right)}$ denote i.i.d. random variables drawn from a standard normal distribution.

To obtain the perceptual model one has to invert the generative model given in Equation (1). The detailed description of the model inversion procedure was originally presented in Mathys et al. (2011) and further extended to an arbitrary number of hierarchical levels in Mathys et al. (2014). The authors proposed a method for obtaining simple, delta-rule like, update equations for the perceptual model using the variational approximation. Here we will only show the final set of the update equations that map percepts to posterior beliefs about the current state of the world (for details see Section 4). The update equations for posterior beliefs can be written as

(2) $$\begin{array}{l} \;\mu_{t}^{\left(1\right)}=\mu_{t-1}^{\left(1\right)}+\epsilon_{t}^{\left(1\right)};\;\epsilon_{t}^{\left(1\right)}=\alpha_{t}^{\left(1\right)}\left[o_{t}-\mu_{t-1}^{\left(1\right)}\right],\\ \;\alpha_{t}^{\left(1\right)}=\frac{\sigma_{t}^{\left(1\right)}}{s};\;\frac{1}{\sigma_{t}^{\left(1\right)}}=\frac{1}{s}+\frac{1}{\sigma_{t-1}^{\left(1\right)}+e^{\mu_{t-1}^{\left(2\right)}}},\\ \;\mu_{t}^{\left(2\right)}=\mu_{t-1}^{\left(2\right)}+\epsilon_{t}^{\left(2\right)};\;\epsilon_{t}^{\left(2\right)}=\alpha_{t}^{\left(2\right)}\delta_{t}^{\left(2\right)};\;\alpha_{t}^{\left(2\right)}=\frac{\sigma_{t}^{\left(2\right)}}{2}w_{t}^{\left(2\right)},\\ \frac{1}{\sigma_{t}^{\left(2\right)}}=\frac{1}{\sigma_{t-1}^{\left(2\right)}+\eta }+\frac{w_{t}^{\left(2\right)}}{2}\left(w_{t}^{\left(2\right)}+r_{t}^{\left(2\right)}\cdot \delta_{t}^{\left(2\right)}\right),\\ \;\delta_{t}^{\left(2\right)}=\left[\frac{\sigma_{t}^{\left(1\right)}+{\left(\epsilon_{t}^{\left(1\right)}\right)}^{2}}{\sigma_{t-1}^{\left(1\right)}+e^{\mu_{t-1}^{\left(2\right)}}}-1\right],\\ w_{t}^{\left(2\right)}=\frac{e^{\mu_{t-1}^{\left(2\right)}}}{\sigma_{t-1}^{\left(1\right)}+e^{\mu_{t-1}^{\left(2\right)}}};\;r_{t}^{\left(2\right)}=\frac{e^{\mu_{t-1}^{\left(2\right)}}-\sigma_{t-1}^{\left(1\right)}}{\sigma_{t-1}^{\left(1\right)}+e^{\mu_{t-1}^{\left(2\right)}}}, \end{array}$$

where $\mu_{t}^{\left(1\right)}$ denotes posterior expectations of the hidden state $x_{t}^{\left(1\right)}$, $\epsilon_{t}^{\left(1\right)}$ precision-weighted prediction error, $\sigma_{t}^{\left(1\right)}$ posterior uncertainty, and $\alpha_{t}^{\left(1\right)}$ the learning rate at the first level of the hierarchy. Equivalently, $\mu_{t}^{\left(2\right)}$ denotes posterior expectations of the hidden state $x_{t}^{\left(2\right)}$, $\epsilon_{t}^{\left(2\right)}$ precision weighted prediction error, $\sigma_{t}^{\left(2\right)}$ posterior uncertainty, and $\alpha_{t}^{\left(2\right)}$ the learning rate at the second level of the hierarchy. This implementation of the perceptual model has six free parameters $\left\{\mu_{0}^{\left(1\right)},\sigma_{0}^{\left(1\right)},s,\mu_{0}^{\left(2\right)},\sigma_{0}^{\left(2\right)},\eta \right\}$.

*Change Point Models* are based on the assumption that the environment goes through periods of stability intermixed with sudden and unpredictable changes in the hidden states of the world. The original Bayes-optimal solution for the change-point detection problem (Adams and MacKay, 2007) was adapted and simplified in Nassar et al. (2010) in order to provide a model that better describes human perception and decision making in changing environments. In these simplified models each observation *o*_*t*_ is used to estimate the probability that a change in the world occurred, where the change-point probability modulates the learning rate; the more likely is the change, the higher the learning rate will be. Here we will reformulate the generative model used by Nassar et al. (2010) as a switching-state-space model (see Section 4 for details), which allows for a more general way of modeling sudden transitions in the state of the world. By using the switching-state-space formulation of the generative model we can derive the corresponding perceptual model by applying the variational approximation to model inversion. The advantage of this formulation is that the update equations obtained via variational approximation are directly comparable to the update equations of the HGF perceptual model (Equation 2). Crucially, the update equations obtained from the variational approximation are almost identical to the update equations originally proposed in Nassar et al. (2010).

A generative model of a simple switching-state-space process is defined as

(3) $$\begin{array}{l} x_{t}^{\left(1\right)}=\{\begin{array}{ll} x_{t-1}^{\left(1\right)}+\sqrt{w_{1}}\cdot n_{t}^{\left(1\right)}, & \text{with probability }1-h,\\ \sqrt{w_{2}}\cdot n_{t}^{\left(1\right)} & \text{with probability }h \end{array}\\ \;o_{t}=x_{t}^{\left(1\right)}+\sqrt{s}\cdot n_{t}^{\left(o\right)}. \end{array}$$

In analogy to the HGF in Equation (2), we will obtain the perceptual model by applying variational Bayesian approximation (for details see Section 4). The update equations of the posterior beliefs are written as

(4) $$\begin{array}{l} \mu_{t}^{\left(1\right)}=\mu_{t-1}^{\left(1\right)}+\epsilon_{t}^{\left(1\right)};\;\epsilon_{t}^{\left(1\right)}=\alpha_{t}^{\left(1\right)}\left[o_{t}-\mu_{t-1}^{\left(1\right)}\right],\\ \alpha_{t}^{\left(1\right)}=\frac{\sigma_{t}^{\left(1\right)}}{s};\;\frac{1}{\sigma_{t}^{\left(1\right)}}=\frac{1-\Omega_{t}}{\sigma_{t-1}^{\left(1\right)}+w_{1}}+\frac{1}{s},\\ \;\Omega_{t}=\frac{N\left(o_{t};0,s+w_{2}\right)\cdot h}{\begin{array}{l} N\left(o_{t};\mu_{t-1}^{\left(1\right)},\sigma_{t-1}^{\left(1\right)}+w_{1}+s\right)\cdot (1-h)\\ \;+\;N\left(o_{t};0,s+w_{2}\right)h \end{array}},\\ \mu_{t}^{\left(2\right)}\propto \ln\frac{\Omega_{t}}{1-\Omega_{t}};\;\epsilon_{t}^{\left(2\right)}\propto \mu_{t}^{\left(2\right)}-\mu_{t-1}^{\left(2\right)}, \end{array}$$

As in Equation (2), the subscript *t* denotes discrete time points, μ^(1)^ denotes posterior expectations (of *x*^(1)^), ϵ^(1)^ precision weighted prediction error, σ^(1)^ posterior uncertainty, and α^(1)^ learning rate at the first level of the hierarchy. Note that the change point probability Ω_*t*_ has a similar effect on the learning rate $\alpha_{t}^{\left(1\right)}$ as the volatility term $\mu_{t}^{\left(2\right)}$ of the HGF model. Hence, we defined a re-parametrization of the change point probability Ω_*t*_ as

(5) $$\Omega_{t}=\frac{1}{1+e^{-a\cdot \mu_{t}^{\left(2\right)}}};\;a>0,$$

where *a* denotes an arbitrary scaling constant. This parametrization of Ω_*t*_ allows a direct comparison of the change point probability with the posterior estimates of volatility $\mu_{t}^{\left(2\right)}$ in the HGF model. This implementation of the perceptual models has also six free parameters $\left\{\mu_{0}^{\left(1\right)},\sigma_{0}^{\left(1\right)},s,w_{1},w_{2},h\right\}$.

Note that in both perceptual models in Equations (2) and (4) the update of posterior expectations about the current state of the world is regulated by adaptive learning rates. In other words, the influence of the prediction errors on the change of the expectations is adaptive and proportional to the posterior uncertainty about the current expectations. This adaptive learning is the key feature of both perceptual models that allows for fast changes of beliefs in dynamic environments.

### 2.2. Simulation of experimental environments

To investigate the properties of the two perceptual models (CPM and HGF) we will emulate typical experiments in which participants are instructed to report their estimate of the true state of an environmental feature (e.g., the true position of the dot on a screen, the true orientation of the bars in an image; Behrens et al., 2007; Summerfield et al., 2011; Nassar et al., 2012; Iglesias et al., 2013; Diaconescu et al., 2014; McGuire et al., 2014; Paliwal et al., 2014; Vossel et al., 2014, 2015), where the estimate is obtained from a series of noisy observations.

**Figure 2:**
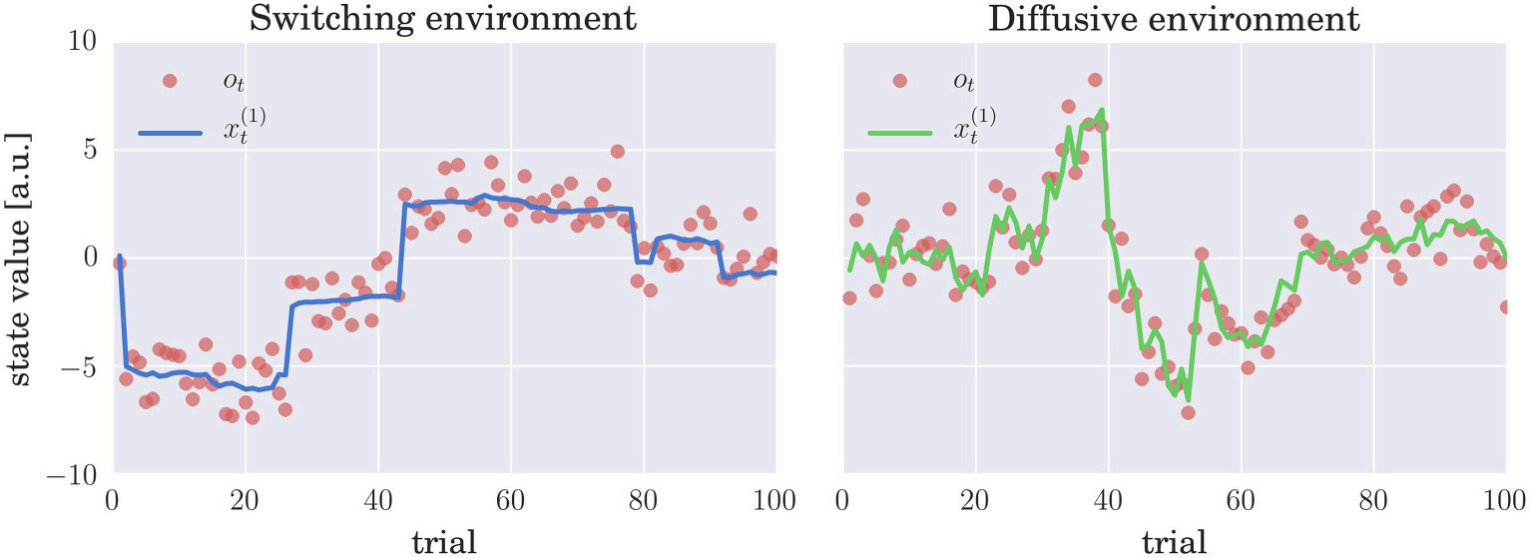
**Evolution of hidden states and sensory stimuli: Typical trajectories of the hidden states $x_{t}^{\left(1\right)}$ (thick lines) and the stimuli (observations) *o*_*t*_ (red circles) in (left) switching and (right) diffusive environment**. In the switching environment the hidden state $x_{t}^{\left(1\right)}$ either follows (with probability *h* = 0.9) a Gaussian random walk (diffusion rate *w*_1_ = 0.01) or corresponds to an i.i.d. random variable (with probability *h* = 0.1) drawn from a zero mean Gaussian distribution with variance *w*_2_ = 10; see Equation (3) for details. In the diffusive environment the $x_{t}^{\left(1\right)}$ follows a Gaussian random walk with diffusion rate $d_{t}=e^{x_{t}^{\left(2\right)}}$, where $x_{t}^{\left(2\right)}$ denotes volatility. Importantly, $x_{t}^{\left(2\right)}$ also follows a Gaussian random walk with diffusion rate η = 0.1. In both environments the observations are generated by adding a standard normal deviate to $x_{t}^{\left(1\right)}$, that is, the observation are corrupted by the observation noise.

As in the past experimental studies (e.g., Behrens et al., 2007; Summerfield et al., 2011; Nassar et al., 2012; Iglesias et al., 2013; McGuire et al., 2014; Vossel et al., 2014), we will introduce two sources of uncertainty in our simulated environments. First, the observations will be distorted by an observation noise, i.e., a standard normal deviate $n_{t}^{\left(o\right)}$ is added to the true value $x_{t}^{\left(1\right)}$ of the hidden state, see Equations (1) and (3). Second, the true state of the world $x_{t}^{\left(1\right)}$ is subject to change, where we will consider two mechanisms for generating the state change, which correspond to the generative processes of the CPM and HGF models. Hence, we will emulate two distinct environments: (i) A switching environment in which at every time step the state of the world can suddenly change with a low probability or follow, with a high probability, a Gaussian random walk (see Equation 3 and Figure 2 for an example of a typical trajectory of the hidden state). (ii) A diffusive environment in which the state of the world follows a Gaussian random walk, in which the diffusion rate depends on a volatility variable, which itself follows a random walk (see Equation 1 and Figure 2 for an example of a typical trajectory of the hidden state).

Using these two experimental environments for the analysis of behavioral models allowed us to test: (i) whether the performance of the simulated agent (participant) depends on the mechanism used to generate sensory stimuli; (ii) whether the accuracy of the model comparison and parameter inference depends on the experimental environment used to generate the sensory stimuli. If there are differences in accuracy, knowing these may be useful for future experiments to generate stimuli that enable the best available accuracy of model comparison.

In summary, the present, synthetic experimental study follows a 2 × 2 factorial design with two experimental environments (switching vs. diffusive) and two synthetic agents (CPM and HGF) placed in these environments.

### 2.3. Simulating participants behavior

In a behavioral experiment participants typically first go through a training session. The training allows participants to sufficiently familiarize with the task so that any learning that might occur during the main experimental session can be neglected. In other words, participants have the opportunity to adjust their internal model of the world to the experimental environment.

To emulate this training session we first optimized the free parameters of the two perceptual models in both the switching and the diffusive environment and then tested model performance in the experimental sessions (newly generated stimuli) of fixed duration. In real experiments, experimenters typically assume that the environment has been learned by participants accurately enough to not have an impact on inference. Following this approach, we aimed at ensuring that model comparison is done in the ideal setting that the environments are learned appropriately. Therefore, we performed a parameter optimization over a large number of experimental blocks, i.e., we performed a very long training session. In this way we ensured that the high accuracy of model comparison is not caused by residual suboptimalities in representing the task environment, which may induce large differences in the behavior generated by the two models.

**Figure 3:**
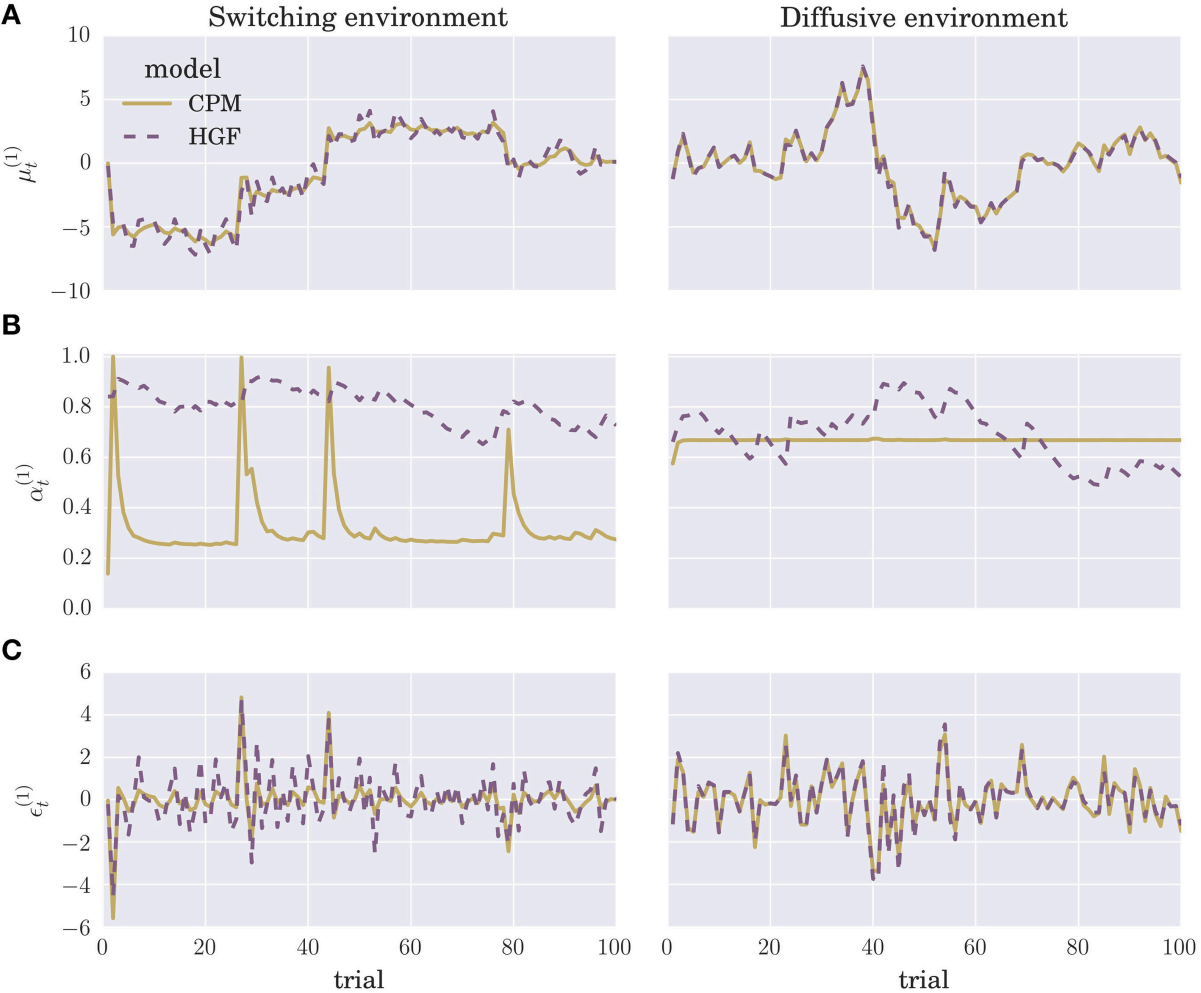
**Evolution of internal model variables: Typical trajectories of (A) posterior expectations $\mu_{t}^{\left(1\right)}$, (B) learning rates $\alpha_{t}^{\left(1\right)}$, and (C) precision-weighted prediction errors $\epsilon_{t}^{\left(1\right)}$ for the CPM (yellow line) and HGF (dashed violet line) perceptual model (for details see Equation 4 and Equation 2)**.

We optimized the parameters of each perceptual model by maximizing the log marginal likelihood—i.e., minimizing the surprise about the sensory stimuli—over a training session consisting of *N* = 1000 experimental blocks, where each block contained *T* = 100 trials (see Section 4 for details). To give the reader an intuition about the differences in the time evolution of posterior beliefs and the prediction errors of the two optimized perceptual models, we show in Figure 3 the trajectories of the internal variables at the first level of the hierarchy (posterior expectation μ^(1)^ Figure 3A, learning rate α^(1)^ Figure 3B, and precision weighted prediction error ϵ^(1)^ Figure 3C). Although there are obvious differences in the learning rate trajectories α^(1)^ of the two perceptual models, the posterior expectations μ^(1)^ are highly correlated between different models. Hence, the two perceptual models will generate very similar sequences of behavioral responses, if we make behavioral responses directly proportional to posterior expectations (see below). The more similar is the sequence the more difficult will be the classification of the behavior.

To quantify the similarity between the posterior expectations obtained from the CPM and HGF models we estimated the performance of both models in each of the two environments. We defined the model performance as the root-mean-square error (RMSE) of the posterior beliefs $\mu_{t}^{\left(1\right)}$ from the true hidden state of the environment $x_{t}^{\left(1\right)}$, hence

(6) $$P_{i}^{2}=\frac{1}{T}\sum_{t=1}^{T}{\left(\mu_{i,t}^{\left(1\right)}-x_{t,i}^{\left(1\right)}\right)}^{2},\;i\in \{1,\ldots ,N\}.$$

Note that the better the model is performing the lower is the RMSE *P*_*i*_ of the *i*th experimental block.

**Figure 4:**
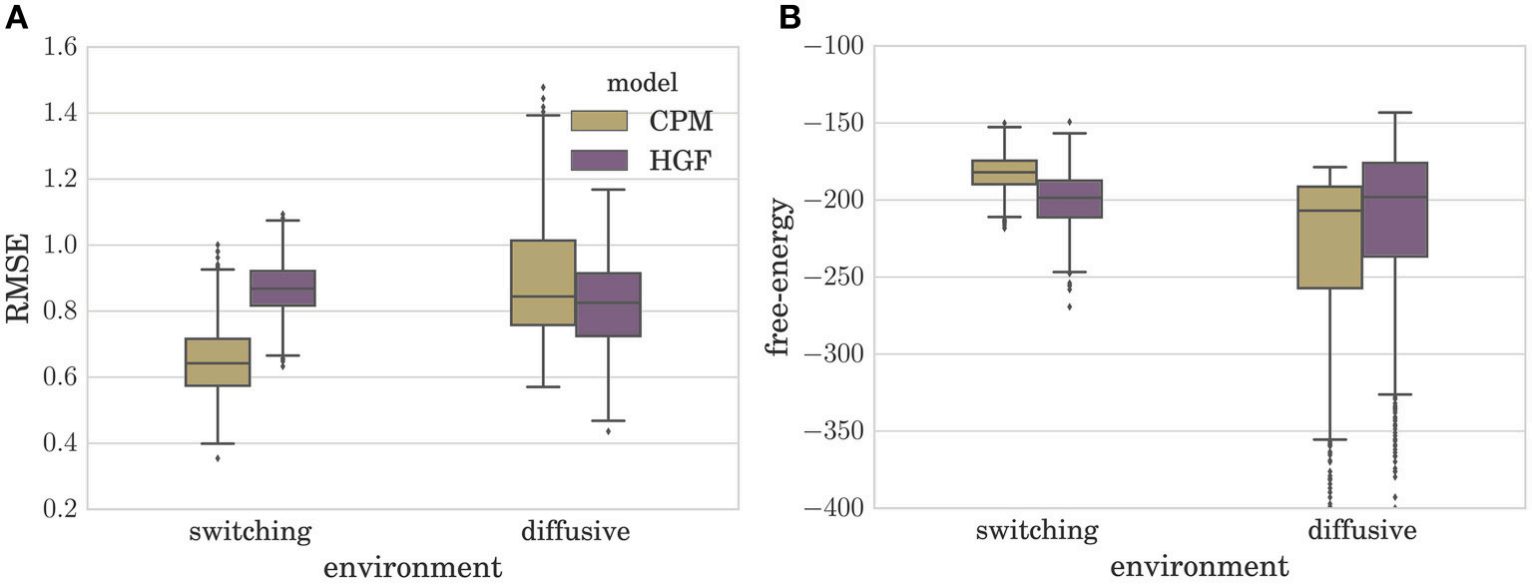
**Boxplot of the distribution of (A) the root-mean-squared error (RMSE) defined in Equation (6), and (B) variational free-energy defined in Equation (30)**. Both quantities were estimated over *N* = 1000 simulated experiments for each type of perceptual model (CPM and HGF) with optimized parameters. Note that the lower the RMSE is the better is the performance of the behavioral model. The boxes span the range from the 25th to the 75th percentile, black horizontal lines denote the median, and whiskers span the range from 1.5 of the inter-quartile (IQR) range below the low quartile to the 1.5 IQR above the upper quartile. Diamonds indicate the outliers.

In Figure 4A we depict the distribution of RMSEs estimated over a set of *N* = 1000 simulated experiments (*T* = 100), for both the CPM and the HGF, in the two environments. Note that the type of perceptual model with better performance in a given environment corresponds to the model that matches the generative mechanism of observations used in that environment (e.g., CPM performs best in the switching environment). Interestingly, as can be seen from Figure 4A, the difference between median model performance in the diffusive environment is not as large as the equivalent difference in the switching environment. This indicates that the CPM is better at adapting to slow changes than the diffusive model is in adapting to fast changes. The same can be inferred from the free-energy distribution (approximate marginal log-likelihood of the perceptual model; see Section 4 for details) shown in Figure 4B. Note that in the switching environment the median free-energy is higher for the CPM (hence the surprise of the CPM model about the sensory stimuli is lower), whereas in the diffusive environment the median free-energy is higher for the perceptual model based on the HGF. This relationship between performance and free-energy is expected: As the variational free-energy provides the lower bound on the marginal log-likelihood (see Section 4), one expects a higher likelihood (lower surprise)—hence better performance—for the sensory stimuli that was generated from the same process that defines the corresponding perceptual model.

To complete the generative model of behavior, we will assume that behavioral response *r*_*t*_ at trial *t* reflects the posterior expectation about the hidden state of an environmental feature corrupted by some response noise, thus

(7) $$r_{t}=\mu_{t}^{\left(1\right)}+\sqrt{\sigma_{r}}n_{t}^{\left(r\right)};\;n_{t}^{\left(r\right)}\sim N\left(0,1\right),$$

where σ_*r*_ denotes the variance of the response noise. Note that this response model can be derived as an optimal response under a quadratic loss function. This assumption is often used in sensorimotor control and learning (Körding and Wolpert, 2004). To generate behavioral responses we will always use the optimized parameter values (for each perceptual model and in each environment) that were estimated from the long training session.

As our goal is to test how accurately we can infer the free model parameters for each model and how accurately we can infer the trajectories of the internal perceptual variables (e.g., $\mu_{t}^{\left(1\right)}$, $\alpha_{t}^{\left(1\right)}$, *etc.*) of the perceptual models, we will simulate two levels of response noise variance σ_*r*_; a low noise level σ_*r*_ = 1 and a high noise level σ_*r*_ = 5. In this way we can test the influence of large response noise on the accuracy of the model comparison, the inference of free model parameters, and the reconstruction of the trajectories of the perceptual variables.

### 2.4. Response likelihood

If in the simulated experiment we measure *T* observations and the same number of responses, by using Equation (7) we can define the response likelihood as

(8) $$p\left(R_{T}|O_{T},\theta_{m},\sigma_{r}\right)=\prod_{t=1}^{T}N\left(r_{t};\mu_{t}^{\left(1\right)}\left(\mu_{t-1}^{\left(1\right)},o_{t},\theta_{m}\right),\sigma_{r}\right),$$

where *R*_*T*_ denotes the set of measured responses during an experimental block of duration *T*, *O*_*T*_ denotes the set of stimuli (observations) presented to a simulated participant, and θ_*m*_ the set of free parameters of the corresponding perceptual model, hence *m* ∈ {*CPM, HGF*}.

Although we consider here only a simple version of the response model, it would be straightforward to extend it to more complex situations and to consider a comparison between different variants of the response model (see Section 3 for details).

### 2.5. Inference of free parameters

Here we will test the reliability of the inference (estimate) about the free parameters of the two behavioral models (see Table 1) using two different approaches. We will compare one of the most commonly used methods for fitting behavioral models, the maximum likelihood estimation (MLE) (Behrens et al., 2007; Wilson et al., 2013), to the Bayesian inference (BI) method (Box and Tiao, 1992; Daunizeau et al., 2010b; Iglesias et al., 2013).

**Table 1:** **Free parameters of the two behavioral models: Hierarchical Gaussian Filter (HFG) and Change Point Model (CPM), see Equations (2) and (4) for details**.

**Model type**	**Perceptual parameters**	**Response parameters**
HGF	$\mu_{0}^{\left(1\right)},\sigma_{0}^{\left(1\right)},s$, $\mu_{0}^{\left(2\right)},\sigma_{0}^{\left(2\right)},\eta$	σ_*r*_
CPM	$\mu_{0}^{\left(1\right)},\sigma_{0}^{\left(1\right)},s$, *w*_1_, *w*_2_, *h*	σ_*r*_

The MLE approach is based on finding the set of parameter values that maximize the (log)likelihood of a behavioral model (Equation 8). The maximum likelihood method is known to fail when applied to the estimation of unknown parameters of dynamical systems (Horbelt, 2001; Judd, 2007), as in these cases the likelihood function typically has a complex multi-modal form, which makes a numerical search for global maxima highly extensive. In contrast, the BI based methods have been found to be robust when inferring parameters even of highly non-linear dynamical system and are known to be robust and accurate when used in data analysis (Carlin and Louis, 1997; Woolrich et al., 2009; Daunizeau et al., 2010a; Mathys et al., 2014; Lomakina et al., 2015). Still, the limitation of the BI is that the estimate of the exact posterior parameter distribution (posterior probability of parameter values) is analytically intractable. Hence, it is typically necessary to apply an approximate inference method. Here, we have selected a rather simple approach, the Laplace approximation (LA) that constrains the posterior probability to the normal distribution (Chickering and Heckerman, 1997; Friston et al., 2007). Nevertheless, in spite of its simplicity, we will demonstrate here several advantages, both for parameter inference and model comparison, of the selected Bayesian Inference-Laplace approximation (BI-LA) compared to the MLE method. The details of both approaches can be found in the Section 4 section.

**Figure 5:**
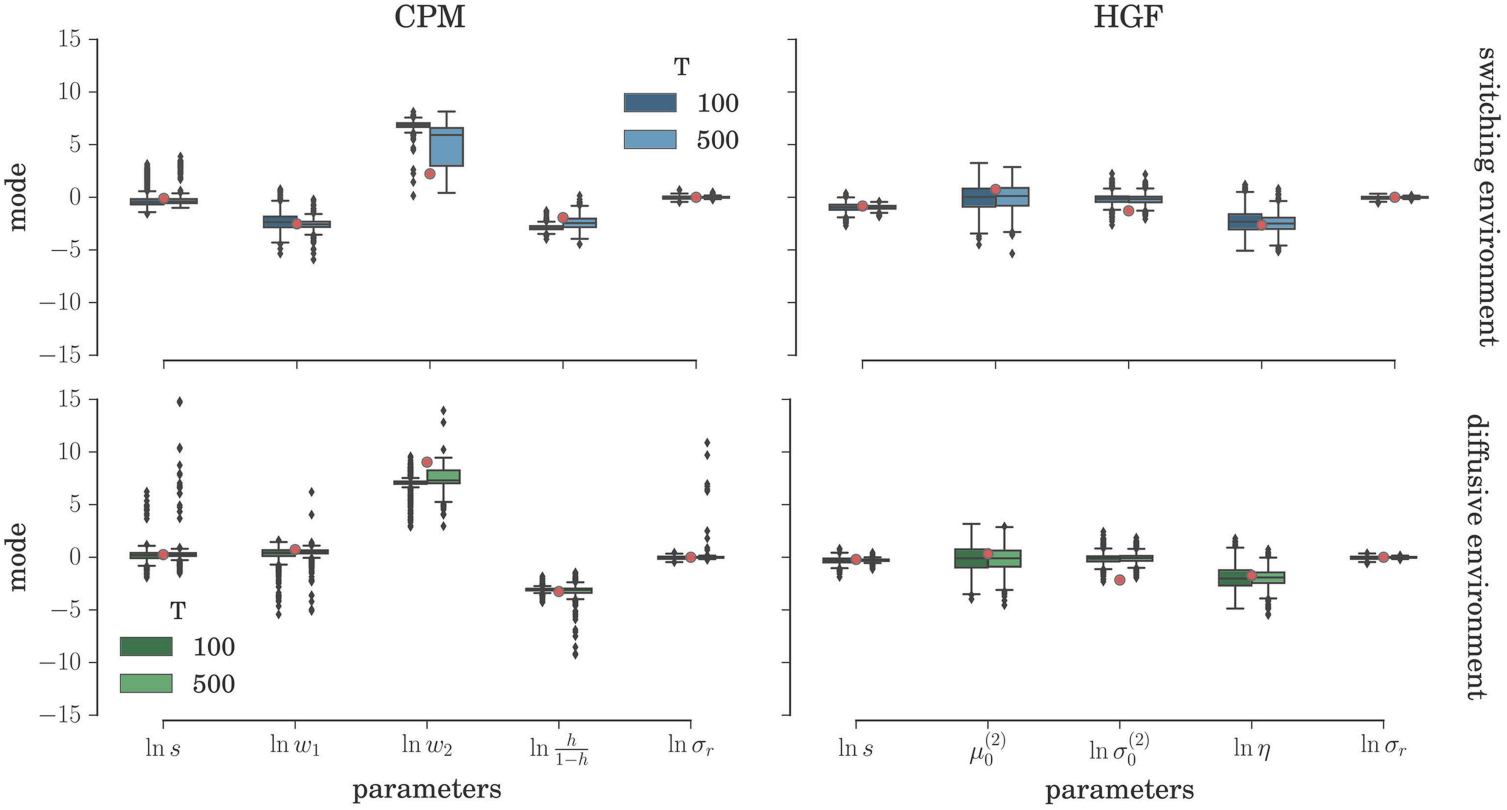
**Parameter inference with the BI-LA method: Boxplot of the distribution of the mode of the posterior free parameter probability (see Equation 58) estimated over *n* = 1000 synthetic agents in the low response noise condition (σ_*r*_ = 1)**. The red circles indicate the true parameter value. The boxes span the range from the 25th to the 75th percentile, black horizontal line within each box shows the median, and whiskers span the range from 1.5 of the inter-quartile (IQR) range below the low quartile to the 1.5 IQR above the upper quartile. Diamonds indicate the outliers.

**Figure 6:**
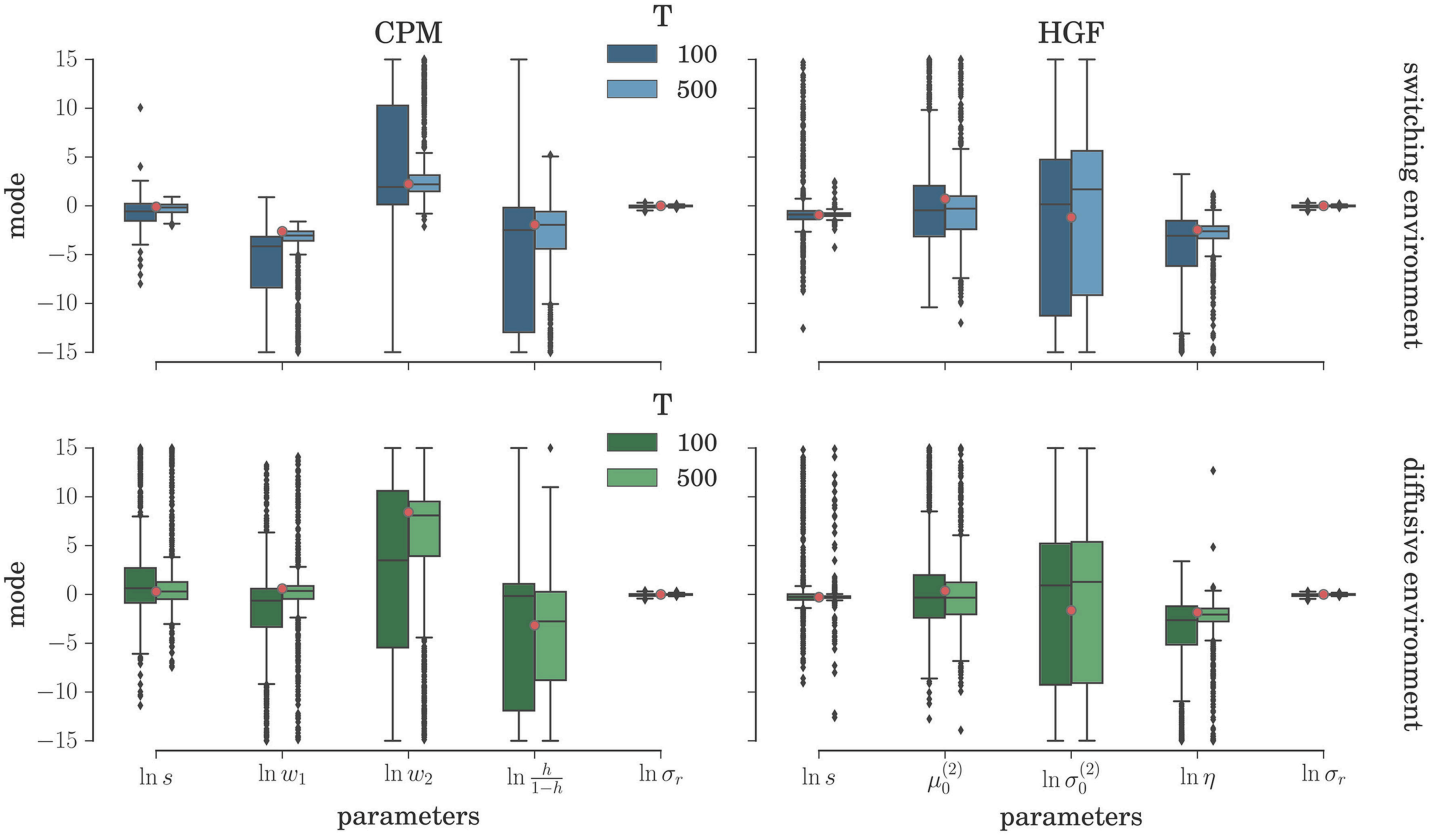
**Parameter estimation with the MLE method: Boxplot of the distribution of the maximum likelihood parameter estimate (see Equation 61) estimated over *n* = 1000 synthetic agents in the low response noise condition (σ_*r*_ = 1)**. The red circles indicate the true parameter value. The boxes span the range from the 25th to the 75th percentile, black horizontal line within each box shows the median, and whiskers span the range from 1.5 of the inter-quartile (IQR) range below the low quartile to the 1.5 IQR above the upper quartile. Diamonds indicate the outliers.

To comprehensively test the reliability of the two inference schemes we have simulated the behavioral responses of *n* = 1000 synthetic agents (i.e., simulated participants) for each behavioral model and the response noise level. Each of these *n* agents was exposed to four experimental conditions (two different environments, and two different durations of the experiment). The full set of free parameters of both perceptual models is shown in Table 1. Note that in both cases the complete behavioral model (the perceptual model plus the response model) has in total seven parameters. In Figure 5 we present the distribution of the posterior mode for five out of seven parameters (we exclude the prior expectation μ_0_ and uncertainty σ_0_ for clarity, the distribution for all parameters is shown in Figure S1) for the low response noise level (σ_*r*_ = 1) and different experiment durations *T*. Importantly, we did not find any obvious differences in the accuracy of the parameter estimation between the low and the high noise levels (see Figures S1, S3). The distribution of the maximum likelihood parameter values obtained using MLE are shown in Figure 6 (see Figure S2 for the complete set of plots). One obvious result is that the median values obtained under both the BI-LA and MLE method are not too different from the true values. However, the variability of the expected parameter values is much smaller when using Bayesian inversion scheme compared to the MLE.

**Figure 7:**
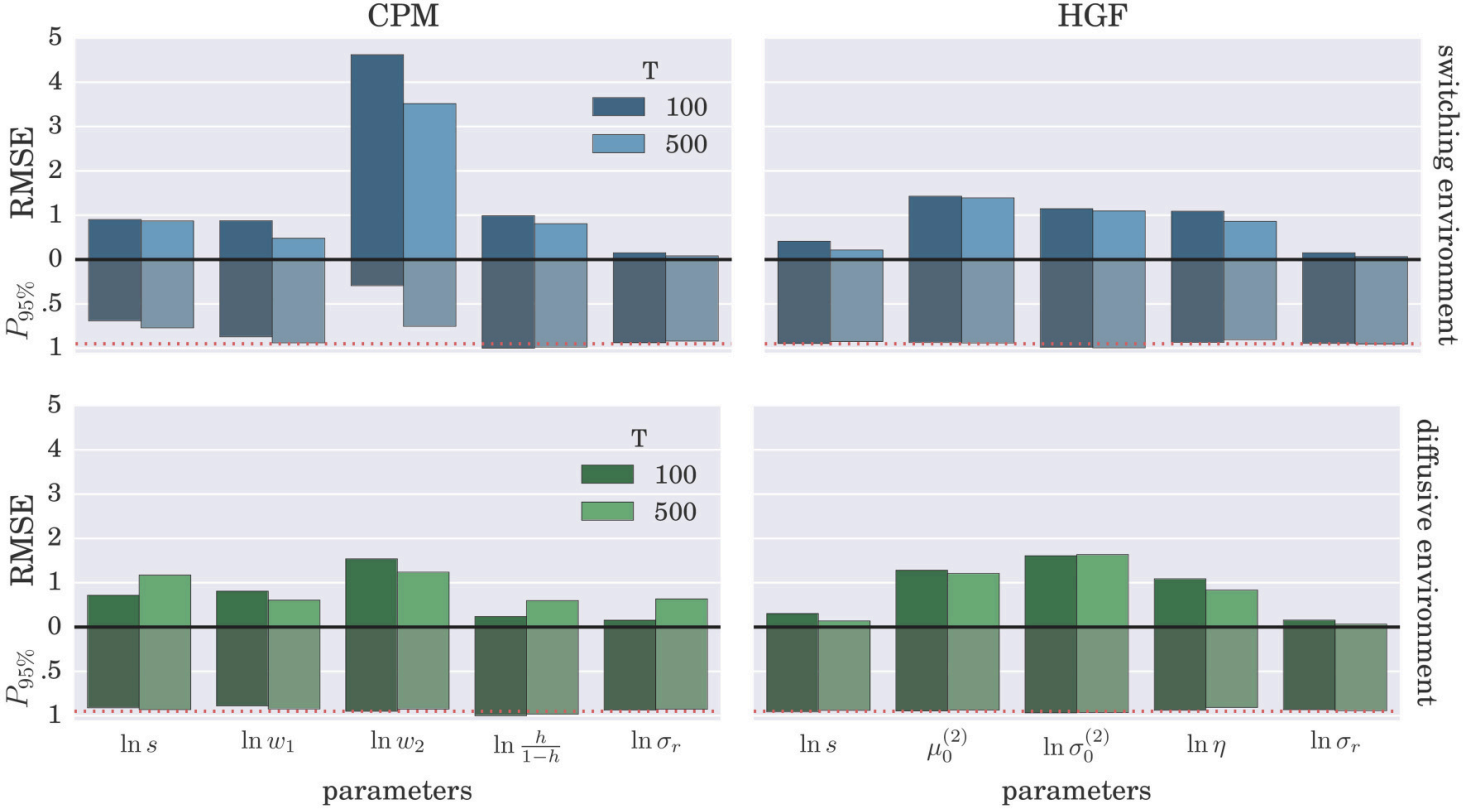
**Estimation accuracy for the BI-LA method: Colored bars above the zero line (black solid line) show the root-mean-square error (RMSE) of the posterior mode over *n* = 1000 synthetic agents in the low response noise case (σ_*r*_ = 1)**. The bars below the zero line denote the probability *P*_95%_ that the true parameter value is within two standard deviations from the posterior mode. The red dotted line marks the 0.95 probability level.

**Figure 8:**
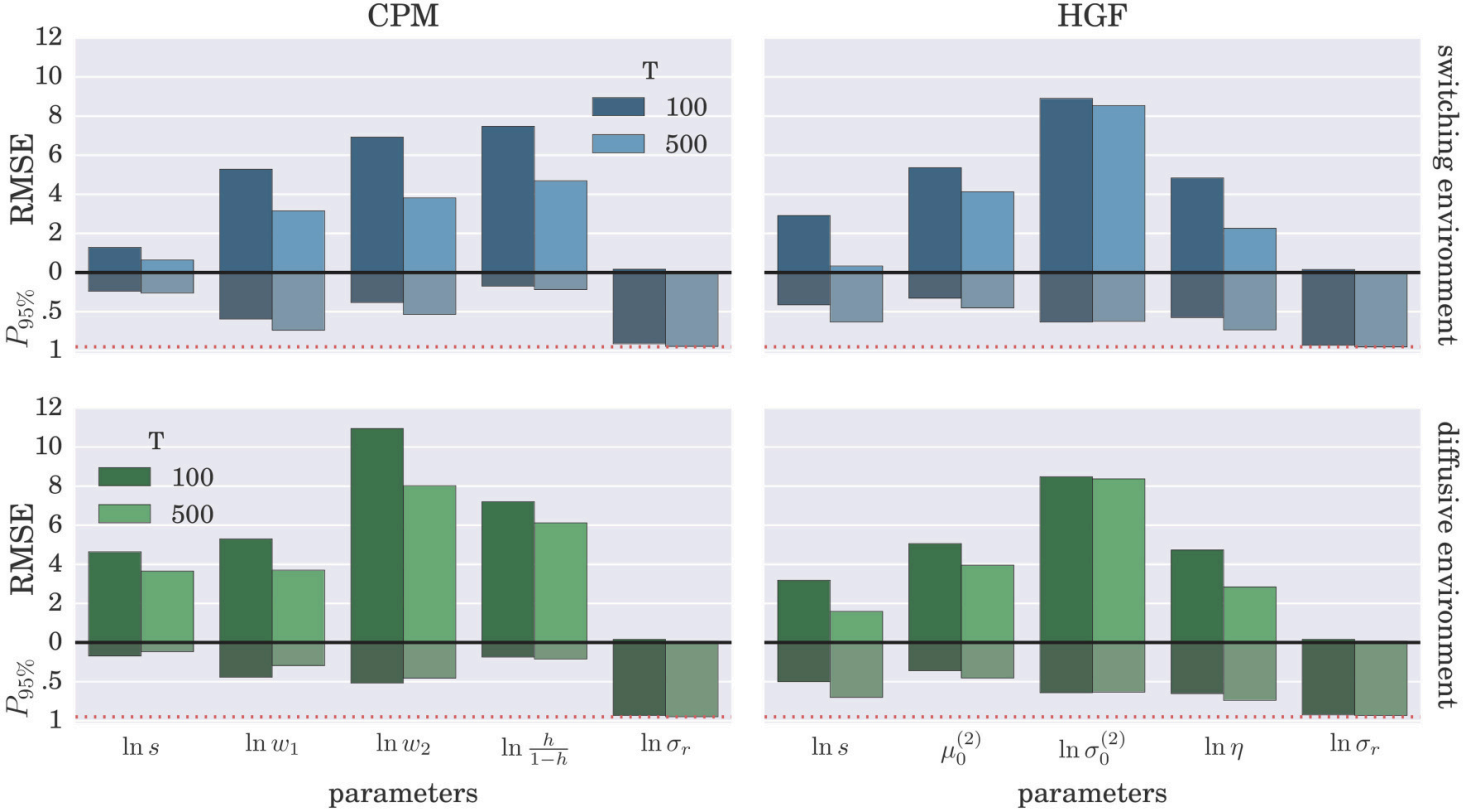
**Estimation accuracy for the MLE method: Colored bars above the zero line (black solid line) show the root-mean-square error (RMSE) of the parameter estimate over *n* = 1000 synthetic agents in the low response noise case (σ_*r*_ = 1)**. The bars below the zero line denote the probability *P*_95%_ that the true parameter value is within two standard deviations from the maximum likelihood value. The red dotted line marks the 0.95 probability level.

To demonstrate further that the BI-LA provides indeed a more accurate estimate, compared to the MLE, we have computed the RMSE for each parameter over the whole set of *n* estimates of the parameter values. In addition, we estimated the probability that the true parameter value falls within the interval spanning ±2·*std* (where std denotes standard deviation) from the estimated value of each model parameter. The results of these “goodness-of-fit” tests for the Bayesian inversion scheme are shown in Figure 7 and for the MLE method in Figure 8. We observe a strong advantage of the BI-LA method compared to the MLE method, as we found across all parameters lower RMSE. Lower RMSE indicates that the estimated parameter value deviates less from the true parameter value when the estimation is performed using the BI-LA method compared to the MLE method. Furthermore, in contrast to the MLE estimate, we find that using the BI-LA method the true parameter value has the measured probability *P*_95%_ ≈ 0.95 to be within the theoretically determined 95% probability interval. As we assume that the posterior parameter distribution corresponds to the normal distribution, this match of the two probabilities indicates that a normal distribution is indeed a good approximation for the posterior parameter probability; with few exceptions (see the Figures S3, S4 for the complete analysis).

### 2.6. Model comparison

In this section, we will test how reliably we can infer the behavioral model that has generated the observed behavior given the known set of sensory stimuli presented to a simulated participant. To perform model selection we have used a well-established method for Bayesian model selection in group studies (Stephan et al., 2009; Rigoux et al., 2014). The method is used to estimate the posterior model probability over a group of participants, given the model log-evidence estimated from behavioral data of each participant within the group. Importantly, this approach takes into account random effects on the estimated model log-evidence; hence it provides robust estimates of the posterior model probability even for approximate and noisy estimates of the model log-evidence.

As the number of participants in behavioral experiments is often only around 20 participants (e.g., Behrens et al., 2007; Summerfield et al., 2011; Vossel et al., 2014), we will assume here that the model comparison is estimated from a group of *k* = 20 participants. We sampled *N* = 10,000 groups of 20 simulated participants (agents) from the same pool of *n* = 1000 simulated participants (per experimental condition) that was used above to assess the accuracy of posterior parameter inference (Figures 5–8). Note that all the behavioral responses of *k* agents are generated by the same generative model. Using the generated behavioral responses of agents—and the known set of sensory stimuli—we estimated the model (log)evidence for each of the two model candidates, using either the MLE or BI approach. When using Bayesian inference we have approximated the model evidence using the Laplace approximation (LA). For the MLE approach we have approximated the model evidence using the Bayesian Information Criterion (BIC). Note that for the MLE approach one obtains the same estimate of the posterior model probability with Aikake's Information Criterion (AIC), as both behavioral models have the same number of free parameters.

**Figure 9:**
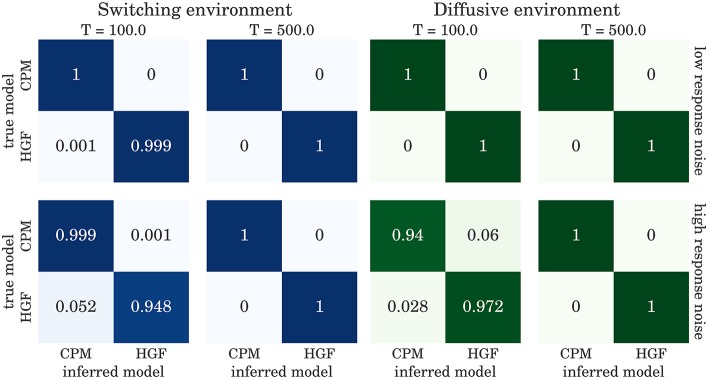
**Confusion matrix for the model inference based on the Laplace approximation of model evidence (see Equation 57): The columns of the matrix represent the inferred behavioral model and the rows of the matrix represent the true behavioral model that has generated the data**.

**Figure 10:**
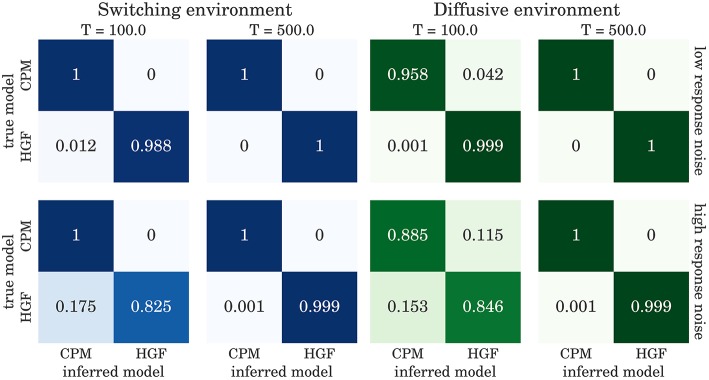
**Confusion matrix for the model inference based on BIC approximation to the model evidence (see Equation 60): The columns of the matrix represent the inferred behavioral model and the rows of the matrix represent the true behavioral model that has generated the data**.

The confusion matrices for model selection are shown in Figure 9, for the BI-LA based model selection, and in Figure 10 for MLE-BIC based model selection. Note that in both environments we find overall more accurate classification of the observed behavior when the model selection is based on BI-LA compared to the one based on the MLE-BIC. For the diffusive environment, we observe that the MLE-BIC based comparison displays lower classification accuracy for low *T* and high response noise. Under the same conditions, the BI-LA based model selection provides higher accuracy.

### 2.7. Correlation analysis

So far we have demonstrated that BI-LA, compared to MLE, provides a more accurate estimation of model parameters and a more accurate model classification across various experimental conditions. However, it is still unclear if the parameter estimation is accurate enough to be relevant for disambiguating between models in a typical model-based neuroimaging analysis. A model-based neuroimaging analysis is used to infer the involvement of different brain areas for a given behavioral task (e.g., Behrens et al., 2007; O'Doherty et al., 2007). Hence, it is essential to accurately estimate the trajectories, over trials, of the internal perceptual variables, such as posterior expectations (μ^(1)^, μ^(2)^) learning rates (α^(1)^, α^(2)^), and precision-weighted prediction errors (ϵ^(1)^, ϵ^(2)^). These internal variables are typically used as parametric regressors in model-based fMRI studies (e.g., Behrens et al., 2007; Iglesias et al., 2013; Vossel et al., 2015). A significant correlation (corrected for multiple comparisons) of blood oxygen level dependent (BOLD) responses in a given brain voxel with any of these regressors would indicate that the activity measured in that voxel represents an aspect of the computational process related to that internal variable.

Thus, the question is, how accurately can we estimate the hidden trajectories of the internal variables of the two perceptual models? To test this we have estimated the correlation coefficients between simulated trajectories and trajectories estimated from the fitted model parameters. The idea here is that a high correlation indicates that the simulated trajectory compares well to the corresponding inferred trajectory.

**Figure 11:**
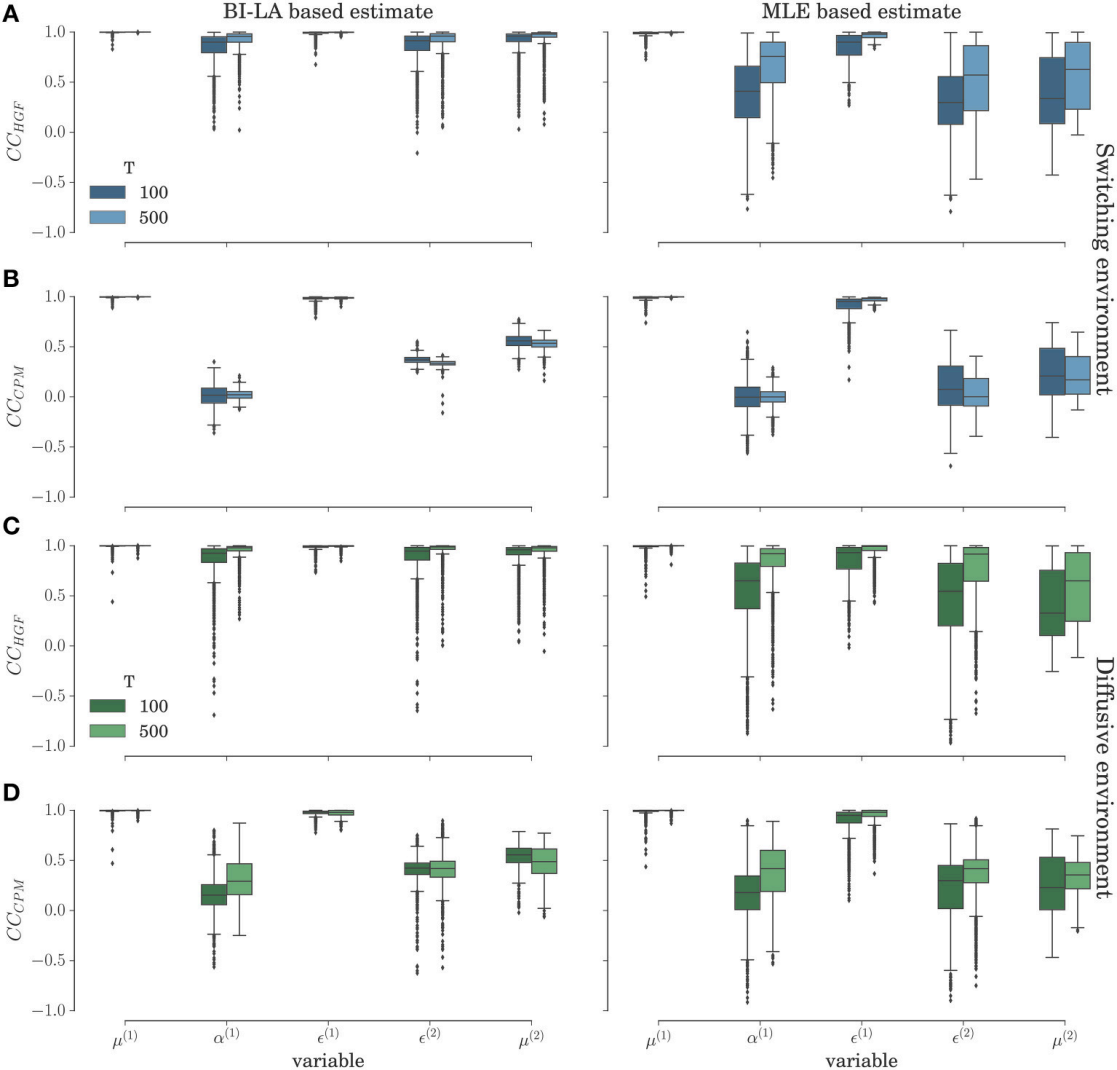
**Correlation analysis in the switching (A–B) and diffusive (C–D) experimental environment for the behavior simulated using the HGF model: Distribution of the correlation coefficient between simulated and inferred trajectories of perceptual variables**. **(A,C)** Correlation coefficient (*CC*_*HGF*_) for the cases when behavior was inferred using the HGF and generated using the HGF; **(B,D)** Correlation coefficient (*CC*_*CPM*_) for the cases when behavior was inferred using the CPM and generated using the HGF. The presented distribution is a combined estimate over the conditions with the low and the high response noise.

In Figure 11 we show the results of the correlation analysis for the behavioral data generated using the HGF model in the two experimental environments. Both plots show the distribution of correlation coefficients estimated over the *n* = 1000 agents in each experimental condition (taken from the same set of behavioral data used in previous sections). The inferred trajectories are obtained by fixing the parameter values to the mode of the posterior distribution or to the MLE. The results show that a high correlation between inferred and simulated trajectories, independent of inference method, is on average achieved only if the correct model is used to estimate the trajectories of internal variables (*e. g.* compare the median value of the correlation coefficient for the learning rate α^(1)^ in Figures 11A,C with the median value for α^(1)^ in Figures 11B,D). This shows that, as with the behavioral analysis, one can expect that a model-based neuroimaging analysis will be sensitive enough to disambiguate between the two models, just based on the usual correlation analysis. However, note that for specific parameters like the posterior expectations μ^(1)^ and precision weighted prediction error ϵ^(1)^ at the first level of the hierarchy, we find high correlations, even if the perceptual model is different from the model that generated behavior. This is expected, because as optimized perceptual models have a rather similar performance (see Figure 4), one would expect posterior expectations and corresponding prediction error to be highly correlated between different perceptual models (see Figures 3A,C). This correlation should also be captured by the inferred trajectories of perceptual variables.

**Figure 12:**
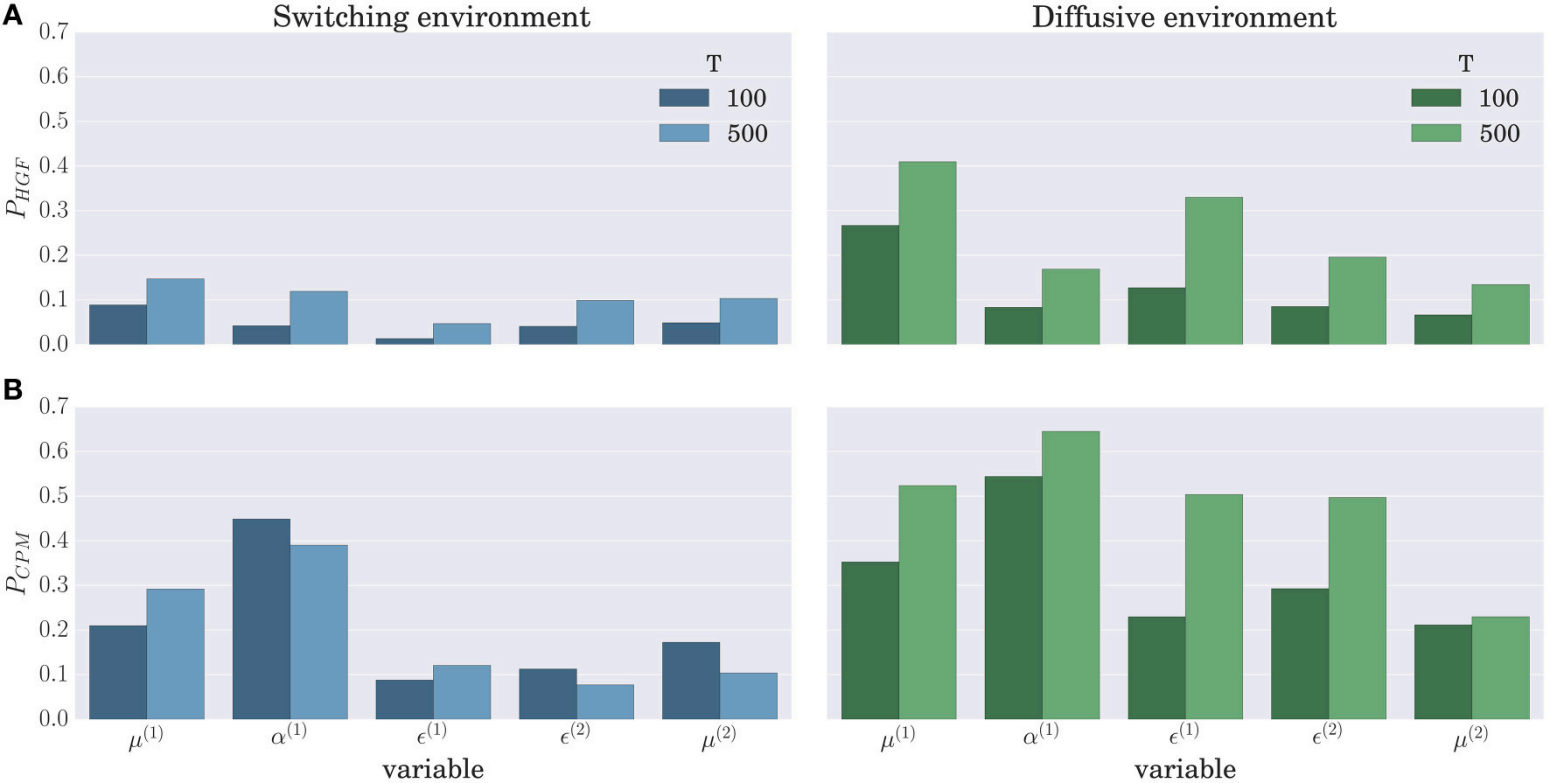
**Probability that the MLE based method provides a higher correlation than the median correlation of the BI-LA based method in the two experimental environments for the behavior simulated using the HGF model: (A) Exceedance probability *P*_*HGF*_ of the MLE based estimate when behavior was inferred using the HGF and generated using the HGF; (B) Exceedance probability *P*_*CPM*_ of the MLE based estimate when behavior was inferred using the CPM and generated using the HGF**. Values below a probability of 0.5 indicate that the BI-LA based median correlation is higher than the MLE based median correlation.

When comparing the BI-LA based estimate of the correlation coefficients with the MLE based estimate (left and right columns of Figure 11) one notices higher median values and lower variances (compare α^(1)^, μ^(2)^, and ϵ^(2)^ in the left and the right column of Figure 11) of the correlation coefficients estimated from the trajectories inferred using the BI-LA approach. To quantify the difference in the estimates of the correlation coefficients caused by the inference method, we have computed the probability that a correlation coefficient of the MLE based estimate is larger than the median value of the correlation coefficient for the BI-LA based estimate. In Figure 12 we present the estimated exceedance probabilities of the correlation coefficients. When the behavioral model that generated behavior and the one used to infer the trajectories of perceptual variables are matched we find consistently higher median correlation values for the BI-LA based estimate (see Figure 12A). Interestingly, a similar but less pronounced relation, is found for the cases when the generating behavioral model and the one used for the inference do not match (see Figure 12B). This suggests that using the BI approach for model-based neuroimaging analysis can be helpful even in the situations when the generative model of percepts is misspecified. Similar conclusions can be drawn from the equivalent correlation analysis results for the behavior generated using the CPM model (see Figures S5, S6).

## 3. Discussion

We performed a comparative analysis of two well-established behavioral models for decision making under uncertainty, in a changing environment. The behavioral models were either based on the Hierarchical Gaussian Filter (HGF) (Mathys et al., 2011, 2014) or on the CPM (Nassar et al., 2010). We have demonstrated that by using an approximate Bayesian inference method and Bayesian model comparison one can substantially improve the estimation accuracy of free model parameters and trajectories of perceptual variables (under the specified range of experimental conditions) when compared to estimation based on MLE. Importantly, when using Bayesian model comparison we can disambiguate between the models with high accuracy independent of the experimental conditions and measurement noise. Furthermore, approximate Bayesian inference provides accurate inference of the free parameters of the behavioral models that define the time evolution of hidden belief states. These results point at the relevance of Bayesian inference methods for computational-experimental research in perceptual decision making and are in agreement with the findings of recent studies that performed similar comparison of different inference methods (Mathys et al., 2014; Lomakina et al., 2015).

Importantly, the methodology presented here is easily extendable to alternative behavioral models, and should be easily applicable to actual experiments. The identification of the precise mechanism that guides human decision making under uncertainty has several important implications for future studies. First, it would improve the confidence in the results and conclusions derived from model based analysis (Nassar and Gold, 2013) of both behavioral and neuroimaging data. Second, the identification of such a mechanism will provide a more robust generalizability of these simple behavioral models to related but more complex tasks (e.g., tasks that require a generalization of behavioral models to higher dimensions). Third, the mechanism of the identified models will provide constraints on the possible neuronal implementation of these phenomenological decision making models.

In what follows we will discuss in more detail possible improvements and extensions to the estimation methods presented here, and potential implications of our findings for the design and model-based neuroimaging data analysis of experimental studies.

### 3.1. Alternative parameter estimation methods

Here the Bayesian estimation of the posterior parameter distribution was based on the Laplace approximation. The Laplace approximation provides one of the computationally most efficient ways to estimate the model evidence and find approximate posterior distribution of free model parameters. However, more elaborate inversion methods do exist, which could provide even higher accuracy in parameter inference. The comparative analysis of several of these inversion schemes has been presented in two recent studies (Mathys et al., 2014; Lomakina et al., 2015), which can be used as a starting point when deciding which of the approximate Bayesian methods to apply to estimation of model parameters and model evidence in a model based analysis of experimental data. The highest accuracy in parameter estimation may be achieved by methods based on Markov Chain Monte Carlo (MCMC). These provide high accuracy at the expense of computational efficiency.

### 3.2. Extensions of model comparison methods

The importance of accurate estimation of model evidence (based on approximate marginal log-likelihood) for accurate model comparison has been stressed before (Penny, 2012). Still, one can think of several modifications which can improve the model selection independent of the approximation method used to estimate the model evidence. Such improvements are especially important for studies that rely on small number of participants, and measure only a small number of trials. Aside from the obvious point of increasing the number of participants, one can measure additional aspects of behavior (e.g., response time and pupil diameter) that can be related to different internal variables of the perceptual model, e.g., the posterior uncertainty $\sigma_{t}^{\left(1\right)}$ or the learning rate $\alpha_{t}^{\left(1\right)}$. Indirect measurements of the internal states that influence the belief process would provide additional constraints on the underlying process that drives behavior, hence it would make, in principle, model selection more accurate. In addition, one could optimize the experimental design in a way that allows for better differentiations of behavioral models (Lewi et al., 2009; Daunizeau et al., 2011). For example, one might present to participants explicitly those sequences of stimuli that one would predict to induce distinct responses between behavioral models. Critically, with the techniques presented here, one can identify, before the experiment, the best candidates for these most revealing stimuli sequences.

### 3.3. Implications of the correlation analysis

The results of correlation analysis (see Figures 11, 12) further stress the relevance of BI methods and model comparison for the neuroimaging studies that rely on model-based analysis of neuroimaging data [e.g., functional magnetic resonance imaging (fMRI), magnetoencephalography (MEG), or electroencephalography (EEG)]. The more accurate is the parameter estimation and model classification, the higher will be the correlation between the inferred belief trajectories and the trajectories that truly generated behavior (provided that the true model is among the models considered). For identifying the computational roles of specific brain areas it is essential that the inferred belief trajectories correlate strongly with the underlying neuronal activity, that is, that the inferred computational process is as close as possible to the computational processes that are implemented in the underlying neuronal network. However, as the trajectories of some of the internal variables at the first level of the hierarchy (posterior expectations μ^(1)^ and precision weighted prediction error ϵ^(1)^) show strong correlation even across different models and for less accurate MLE based approach, one could expect that previous model-based studies that looked for neural correlates of these quantities (e.g., Iglesias et al., 2013; Vossel et al., 2015) would still hold even if an alternative model is shown to be more appropriate. Importantly, we would expect that a subsequent identification of appropriate behavioral model would improve the localization of different aspects of the decision making process to specific brain areas.

### 3.4. Alternative perceptual and response models

Although we focused here on two possible approaches to modeling perception in a changing environment, in practice several extensions and alternative formulations of perceptual models could also be considered: (i) instead of approximate inference one could formulate the update equations using exact inference (Behrens et al., 2007); (ii) the simple Change Point Model presented here could be extended to include arbitrary many learning rate values (Wilson et al., 2013); (iii) representing higher levels of the hierarchy with Dirichlet processes would allow online learning of the discretization levels (Quinn and Karny, 2007; Qian and Aslin, 2014); (iv) adding additional levels of the hierarchy for online learning of the hazard rate and drift rate (Wilson et al., 2010; Mathys et al., 2014).

Beside alternative perceptual models one could also consider extensions to the response model and additional behavioral responses that could be used to further improve the parameter inference and the model comparison in the presence of the response noise. For example, by measuring pupil diameter or asking participants to report their confidence about the response they made, one could relate these quantities to the posterior uncertainty $\sigma_{t}^{\left(1\right)}$ or the learning rate $\alpha_{t}^{\left(1\right)}$ (Nassar et al., 2012). In addition, one could extend the relation between the measured behavioral response and hidden belief states by taking into account possible causes for deviation of behavioral responses from an optimal response (Acerbi et al., 2014). Finally, one of the most interesting extensions of the response models is to include measured neuronal responses within the response model, and use them directly as additional evidence about the underlying computational mechanism (Friston and Penny, 2003; Rosa et al., 2010).

### 3.5. Conclusion

In summary, we have shown that rigorous analysis of behavioral data using Bayesian inference methods can in practice allow us to disambiguate between two well-established models of how humans make decisions in changing and noisy environments, in both behavioral and neuroimaging studies.

## 4. Methods

### 4.1. Derivation of perceptual models

#### 4.1.1. HGF based perceptual model

Starting from the generative model given in Equation (1), we can write the observation likelihood, and the transition probabilities at the two levels of the hierarchy as

(9) $$p\left(o_{t}|x_{t}^{\left(1\right)}\right)=N\left(o_{t};x_{t}^{\left(1\right)},s\right),$$

(10) $$p\left(x_{t}^{\left(1\right)}|x_{t-1}^{\left(1\right)},x_{t}^{\left(2\right)}\right)=N\left(x_{t}^{\left(1\right)};x_{t-1}^{\left(1\right)},{\text{e}}^{x_{t}^{\left(2\right)}}\right)$$

(11) $$p\left(x_{t}^{\left(2\right)}|x_{t-1}^{\left(2\right)}\right)=N\left(x_{t}^{\left(2\right)};x_{t-1}^{\left(2\right)},\eta \right),$$

where N(*x*; μ, σ) denotes a normal distribution of variable *x* with mean μ and variance σ. The full generative model is then obtained as

(12) $$\begin{array}{l} p\left(o_{t},x_{t}^{\left(1\right)},x_{t}^{\left(2\right)},x_{t-1}^{\left(1\right)},x_{t-1}^{\left(2\right)}|O_{t-1}\right)=p\left(o_{t}|x_{t}^{\left(1\right)}\right)p\left(x_{t}^{\left(1\right)}|x_{t-1}^{\left(1\right)},x_{t}^{\left(2\right)}\right)\\ \;p\left(x_{t}^{\left(2\right)}|x_{t-1}^{\left(2\right)}\right)p\left(x_{t-1}^{\left(1\right)},x_{t-1}^{\left(2\right)}|O_{t-1}\right), \end{array}$$

where $p\left(x_{t-1}^{\left(1\right)},x_{t-1}^{\left(2\right)}|O_{t-1}\right)$ denotes prior probability of hidden states given the set of past observation *O*_*t* − 1_ = (*o*_*t*−1_, …, *o*_1_). Integrating out $x_{t-1}^{\left(1\right)}$ and $x_{t-1}^{\left(2\right)}$, from the full generative model, gives us a compact form of the generative model that depends on a predictive distribution, which is obtained as

(13) $$\begin{array}{l} p\left(x_{t}^{\left(1\right)},x_{t}^{\left(2\right)}|O_{t-1}\right)=\\ \;\int \int \text{d}x_{t-1}^{\left(1\right)}\text{d}x_{t-1}^{\left(2\right)}p\left(x_{t}^{\left(1\right)},x_{t}^{\left(2\right)},x_{t-1}^{\left(1\right)},x_{t-1}^{\left(2\right)}|O_{t-1}\right). \end{array}$$

From the compact generative model we write the posterior probability over hidden states as

(14) $$p\left(x_{t}^{\left(1\right)},x_{t}^{\left(2\right)}|O_{t}\right)=\frac{p\left(o_{t},x_{t}^{\left(1\right)},x_{t}^{\left(2\right)}|O_{t-1}\right)}{p\left(o_{t}|O_{t-1}\right)}.$$

As the exact solution for the posterior probability is analytically intractable, we will use the variational approximation to derive the update equations for posterior beliefs shown in Equation (2). The detailed recipe for the derivation procedure can be found in Mathys et al. (2011, 2014), here we will go briefly over the main steps.

In variational inference the joint posterior distribution is simplified with the variational distribution, which assumes that posterior probabilities of different states are independent from each other. Thus, we write

(15) $$p\left(x_{t}^{\left(1\right)},x_{t}^{\left(2\right)}|O_{t}\right)\approx q\left(x_{t}^{\left(1\right)}|\lambda_{t}^{\left(1\right)}\right)q\left(x_{t}^{\left(2\right)}|\lambda_{t}^{\left(2\right)}\right).$$

This factorization allows us to write a lower bound to the marginal log-likelihood as

(16) $$\begin{array}{l} \ln p\left(o_{t}|O_{t-1}\right)\geq {\langle \ln p\left(o_{t},x_{t}^{\left(1\right)},x_{t}^{\left(2\right)}|O_{t-1}\right)\rangle }_{q\left(x_{t}^{\left(1\right)}\right)q\left(x_{t}^{\left(2\right)}\right)}\\ \;+\;H\left[q\left(x_{t}^{\left(1\right)}\right)\right]+H\left[q\left(x_{t}^{\left(1\right)}\right)\right], \end{array}$$

where we omitted $\lambda_{t}^{\left(1\right)}$ and $\lambda_{t}^{\left(1\right)}$ for readability, and where *H*[*q*] denotes differential entropy of *q*. The right hand side of the inequality is called the variational free-energy $F\left(\lambda_{t}^{\left(2\right)},\lambda_{t}^{\left(2\right)}\right)$. By maximizing the free-energy functional with respect to $q\left(x_{t}^{\left(1\right)}|\lambda_{t}^{\left(1\right)}\right)$ and $q\left(x_{t}^{\left(2\right)}|\lambda_{t}^{\left(2\right)}\right)$ one obtains the approximate posterior distribution (Equation 15). Using variational calculus one can demonstrate that the maximum is obtained for

(17) $$\begin{array}{l} q\left(x_{t}^{\left(1\right)}|\lambda_{t}^{\left(1\right)}\right)\propto {\text{e}}^{I\left(x_{t}^{\left(1\right)}\right)},\\ \;I\left(x_{t}^{\left(1\right)}\right)={\langle \ln p\left(o_{t},x_{t}^{\left(1\right)},x_{t}^{\left(2\right)}|O_{t-1}\right)\rangle }_{q\left(x_{t}^{\left(2\right)}|\lambda_{t}^{\left(2\right)}\right)}, \end{array}$$

(18) $$\begin{array}{l} q\left(x_{t}^{\left(2\right)}|\lambda_{t}^{\left(2\right)}\right)\propto {\text{e}}^{I\left(x_{t}^{\left(2\right)}\right)},\\ \;I\left(x_{t}^{\left(2\right)}\right)={\langle \ln p\left(o_{t},x_{t}^{\left(1\right)},x_{t}^{\left(2\right)}|O_{t-1}\right)\rangle }_{q\left(x_{t}^{\left(1\right)}|\lambda_{t}^{\left(1\right)}\right)}, \end{array}$$

where $I\left(x_{t}^{\left(1\right)}\right)$ and $I\left(x_{t}^{\left(2\right)}\right)$ denote variational energy of the hidden states at the first and the second level of the hierarchy, respectively.

We will assume that the normal distribution can be used to represent the approximate posteriors on both levels of the hierarchy, that is, we assume that for accurate inference it is sufficient to keep track only of posterior expectations and uncertainty, hence

(19) $$q\left(x_{t}^{\left(1\right)}|\lambda_{t}^{\left(1\right)}\right)=N\left(x_{t}^{\left(1\right)};\mu_{t}^{\left(1\right)},\sigma_{t}^{\left(1\right)}\right),$$

(20) $$q\left(x_{t}^{\left(2\right)}|\lambda_{t}^{\left(2\right)}\right)=N\left(x_{t}^{\left(2\right)};\mu_{t}^{\left(2\right)},\sigma_{t}^{\left(2\right)}\right).$$

Thus, $\lambda_{t}^{\left(q\right)}=\left(\mu_{t}^{\left(q\right)},\sigma_{t}^{\left(q\right)}\right)$ for *q* ∈ {1, 2}. Having simplified the approximated posterior distribution as a product of normal distributions we can go one step back and compute the predictive distributions (Equation 13) by setting the approximate posterior as a prior in the next time step. Thus, the predictive distributions become

(21) $$p\left(x_{t}^{\left(1\right)}|x_{t}^{\left(2\right)},O_{t-1}\right)=N\left(x_{t}^{\left(1\right)};\mu_{t-1}^{\left(1\right)},\sigma_{t-1}^{\left(1\right)}+{\text{e}}^{x_{t}^{\left(2\right)}}\right)$$

(22) $$\;p\left(x_{t}^{\left(2\right)}|O_{t-1}\right)=N\left(x_{t}^{\left(2\right)};\mu_{t-1}^{\left(2\right)},\sigma_{t-1}^{\left(2\right)}+\eta \right),$$

Hence, the compact form of the generative model is expressed as

(23) $$\begin{array}{l} p\left(o_{t},x_{t}^{\left(1\right)},x_{t}^{\left(2\right)}|O_{t-1}\right)=\\ N\left(o_{t};x_{t}^{\left(1\right)},s\right)N\left(x_{t}^{\left(1\right)};\mu_{t-1}^{\left(1\right)},\sigma_{t-1}^{\left(1\right)}+{\text{e}}^{x_{t}^{\left(2\right)}}\right)\\ N\left(x_{t}^{\left(2\right)};\mu_{t-1}^{\left(2\right)},\sigma_{t-1}^{\left(2\right)}+\eta \right). \end{array}$$

To establish the dependence of posterior beliefs $\lambda_{t}^{\left(1,2\right)}$ on prior beliefs $\lambda_{t-1}^{\left(1,2\right)}$ we substitute Equation (23) into Equation (17) and Equation (18) and obtain

(24) $$\begin{array}{l} q\left(x_{t}^{\left(1\right)}|\lambda_{t}^{\left(1\right)}\right)\propto {\text{e}}^{I\left(x_{t}^{\left(1\right)}\right)},\\ \;I\left(x_{t}^{\left(1\right)}\right)\approx {\langle \ln p\left(o_{t},x_{t}^{\left(1\right)},x_{t}^{\left(2\right)}|O_{t-1}\right)\rangle }_{p\left(x_{t}^{\left(2\right)}|O_{t-1}\right)},\\ \;I\left(x_{t}^{\left(1\right)}\right)\approx -\frac{1}{2}\left[\frac{{\left(o_{t}-x_{t}^{\left(1\right)}\right)}^{2}}{s}+\frac{{\left(x_{t}^{\left(1\right)}-\mu_{t-1}^{\left(1\right)}\right)}^{2}}{\sigma_{t-1}^{\left(1\right)}+{\text{e}}^{\mu_{t-1}^{\left(2\right)}}}\right]+const, \end{array}$$

(25) $$\begin{array}{l} q\left(x_{t}^{\left(2\right)}|\lambda_{t}^{\left(2\right)}\right)\propto {\text{e}}^{I\left(x_{t}^{\left(2\right)}\right)},\\ \;I\left(x_{t}^{\left(2\right)}\right)={\langle \ln p\left(o_{t},x_{t}^{\left(1\right)},x_{t}^{\left(2\right)}|O_{t-1}\right)\rangle }_{q\left(x_{t}^{\left(1\right)}|\lambda_{t}^{\left(1\right)}\right)},\\ \;I\left(x_{t}^{\left(2\right)}\right)\approx -\frac{1}{2}[\frac{\sigma_{t}^{\left(1\right)}+{\left(\mu_{t}^{\left(1\right)}-\mu_{t-1}^{\left(1\right)}\right)}^{2}}{e^{x_{t}^{\left(2\right)}}+\sigma_{t-1}^{\left(1\right)}}\\ \;+\ln\left(e^{x_{t}^{\left(2\right)}}+\sigma_{t-1}^{\left(1\right)}\right)+\frac{{\left(x_{t}^{\left(2\right)}-\mu_{t-1}^{\left(2\right)}\right)}^{2}}{\sigma_{t-1}^{\left(2\right)}+\eta }]+const. \end{array}$$

where $I\left(x_{t}^{\left(1\right)}\right)$ and $I\left(x_{t}^{\left(2\right)}\right)$ denote the variational energy of the hidden states at the first and the second level of the hierarchy, respectively. Note that to avoid the circularity problem we first estimate $I\left(x_{t}^{\left(1\right)}\right)$ given the predictive distribution $p\left(x_{t}^{\left(2\right)}|O_{t-1}\right)$ and then estimate $I\left(x_{t}^{\left(2\right)}\right)$ given the approximate posterior estimate $q\left(x_{t}^{\left(1\right)}|\lambda_{t}^{\left(1\right)}\right)$. In other words, we assume that the beliefs are first updated on the lower level of the hierarchy, and then this information propagates to the level above.

As we have assumed that the approximate posterior distributions $q\left(x_{t}^{\left(1\right)}|\lambda_{t}^{\left(1\right)}\right)$ can be expressed as normal distributions (see Equations 19 and 20) the posterior expectations at each level of the hierarchy are obtained as the modes of the corresponding variational energies, hence

(26) $$\mu_{t}^{\left(q\right)}=\arg\underset{{}_{x_{t}^{\left(q\right)}}}{\max}I\left(x_{t}^{\left(q\right)}\right),$$

where *q* ∈ {1, 2}. As the posterior expectations $\mu_{t}^{\left(1\right)}$ and $\mu_{t}^{\left(2\right)}$ correspond to the maxima of the corresponding variational energy, we can obtain these maxima by applying the Newton's method, hence

(27) $$\mu_{t}^{\left(q\right)}=\rho^{*}-\partial_{x_{t}^{\left(q\right)}x_{t}^{\left(q\right)}}I\left(\rho^{*}\right)\partial_{x_{t}^{\left(q\right)}}I\left(\rho^{*}\right),$$

where ρ^*^ denotes an arbitrary value. By setting ρ^*^ to the corresponding prior expectations $\mu_{t-1}^{\left(1\right)}$ and $\mu_{t-1}^{\left(2\right)}$ and defining posterior uncertainty as

(28) $$\sigma_{t}^{\left(q\right)}={\left[-\partial_{x^{\left(q\right)}x^{\left(q\right)}}I\left(\mu_{t-1}^{\left(q\right)}\right)\right]}^{-1},$$

we obtain the update equations shown in Equation (2).

Finally, having computed the posterior distributions $q\left(x_{t}^{\left(1\right)}|\lambda_{t}^{\left(1\right)}\right)$ and $q\left(x_{t}^{\left(2\right)}|\lambda_{t}^{\left(2\right)}\right)$, we can estimate the marginal log-likelihood of the current observation *o*_*t*_ conditioned on all the past observations *O*_*t* − 1_ as

(29) $$\begin{array}{l} \ln p\left(o_{t}|O_{t-1},\theta \right)\approx F_{t}(\theta )=\int q\left(x_{t}^{\left(2\right)}|\lambda_{t}^{\left(2\right)}\right)I\left(x_{t}^{\left(2\right)}\right)\text{d}x_{t}^{\left(2\right)}\\ \;+\;H\left[q\left(x_{t}^{\left(1\right)}\right)\right]+H\left[q\left(x_{t}^{\left(1\right)}\right)\right]\\ \;\approx -\frac{1}{2}\ln s-\frac{1}{2}\frac{\sigma_{t}^{\left(1\right)}+{\left(o_{t}-\mu_{t}^{\left(1\right)}\right)}^{2}}{s}\\ \;-\frac{1}{2}\ln\left(\sigma_{t-1}^{\left(1\right)}+{\text{e}}^{\mu_{t}^{\left(2\right)}}\right)\\ \;-\frac{1}{2}\frac{\sigma_{t}^{\left(1\right)}+{\left(\mu_{t}^{\left(1\right)}-\mu_{t-1}^{\left(1\right)}\right)}^{2}}{\sigma_{t-1}^{\left(1\right)}+{\text{e}}^{\mu_{t}^{\left(2\right)}}}\\ \;-\frac{1}{2}\ln\left(\sigma_{t-1}^{\left(2\right)}+\eta \right)\\ \;-\frac{1}{2}\frac{\sigma_{t}^{\left(2\right)}+{\left(\mu_{t}^{\left(2\right)}-\mu_{t-1}^{\left(2\right)}\right)}^{2}}{\sigma_{t-1}^{\left(2\right)}+\eta }\\ \;-\frac{1}{2}\ln2\pi +1+\frac{1}{2}\ln\sigma_{t}^{\left(1\right)}\sigma_{t}^{\left(2\right)}. \end{array}$$

where θ denotes the set of fixed parameters of the perceptual model $\left\{\mu_{0}^{\left(1\right)},\sigma_{0}^{\left(1\right)},s,\mu_{0}^{\left(2\right)},\sigma_{0}^{\left(2\right)},\eta \right\}$. For the given set of observations *O*_*T*_, the total surprise (negative marginal log-likelihood) is obtained as

(30) $$-\ln p\left(O_{T}|\theta \right)=-\sum_{t=1}^{T}\ln p\left(o_{t}|O_{t-1},\theta \right)\approx -\sum_{t=1}^{T}F_{t}(\theta ).$$

Hence minimizing surprise about the stimuli presented during an experimental block of length *T* corresponds to maximizing the sum of variational free-energy with respect to set of parameters θ. Starting from some prior parameter *p*(θ) distribution we obtain the optimal parameter values over *N* experimental blocks of length *T* as

(31) $$\theta^{*}=\arg\underset{\theta }{\max}\left[\ln p\left(\theta \right)+\sum_{n=1}^{N}\sum_{t=1}^{T}F_{t}^{\left(n\right)}(\theta )\right].$$

In other words, we assume here that the participants, being exposed to *N* training sessions, would adjust the parameters of their internal representation of the experimental environment in a Bayes optimal fashion.

#### 4.1.2. Change point models

Starting this time from the generative model given in Equation (3), we write the observation likelihood, and transition probabilities at different levels of the hierarchy as

(32) $$\begin{array}{l} \;p\left(o_{t}|x_{t}^{\left(1\right)}\right)=N\left(o_{t};x_{t}^{\left(1\right)},s\right),\\ \;p\left(x_{t}^{\left(1\right)}|x_{t-1}^{\left(1\right)},H_{t}=1\right)=N\left(x_{t}^{\left(1\right)};x_{t-1}^{\left(1\right)},w_{1}\right)\\ \;p\left(x_{t}^{\left(1\right)}|x_{t-1}^{\left(1\right)},H_{t}=2\right)=N\left(x_{t}^{\left(1\right)};0,w_{2}\right)\\ p\left(H_{t}|H_{t-1}\right)=p\left(H_{t}\right)={\left(1-h\right)}^{\delta_{1,H_{t}}}h^{\delta_{2,H_{t}}}. \end{array}$$

For this case the full generative model becomes

(33) $$\begin{array}{l} p\left(o_{t},x_{t}^{\left(1\right)},H_{t},x_{t-1}^{\left(1\right)}|O_{t-1}\right)=p\left(o_{t}|x_{t}^{\left(1\right)}\right)p\left(x_{t}^{\left(1\right)}|x_{t-1}^{\left(1\right)},H_{t}\right)\\ \;p\left(x_{t-1}^{\left(1\right)}|O_{t-1}\right)p\left(H_{t}\right), \end{array}$$

where we omitted the prior probability *p*(*H*_*t* − 1_|*O*_*t* − 1_) as the transition probability *p*(*H*_*t*_|*H*_*t* − 1_) = *p*(*H*_*t*_), that is, the transition probability is independent of prior probability. Similar to the derivations in the previous section, integrating out $x_{t-1}^{\left(1\right)}$ gives us the compact form of the generative model that we use to obtain the posterior probability as

(34) $$p\left(x_{t}^{\left(1\right)},H_{t}|O_{t}\right)=\frac{p\left(o_{t},x_{t}^{\left(1\right)},H_{t}|O_{t-1}\right)}{p\left(o_{t}|O_{t-1}\right)}.$$

Similar to the computations of the posteriors for the HGF, we face here the analytical intractability of the exact posterior distribution. Hence, we will again simplify the posterior distribution with a variational distribution, that is,

(35) $$p\left(x_{t}^{\left(1\right)},H_{t}|O_{t}\right)=q\left(x_{t}^{\left(1\right)}|\lambda_{t}^{\left(1\right)}\right)q\left(H_{t}|\Omega_{t}\right),$$

where

(36) $$q\left(x_{t}^{\left(1\right)}|\lambda_{t}^{\left(1\right)}\right)=N\left(x_{t}^{\left(1\right)};\mu_{t}^{\left(1\right)},\sigma_{t}^{\left(1\right)}\right),$$

(37) $$\;q\left(H_{t}|\Omega_{t}\right)={\left(1-\Omega_{t}\right)}^{\delta_{1,H_{t}}}\Omega_{t}^{\delta_{2,H_{t}}}.$$

We will again use this simplified form of the posterior distribution to define the predictive distributions as

(38) $$p\left(x_{t}^{\left(1\right)}|H_{t}=1,O_{t-1}\right)=N\left(x_{t}^{\left(1\right)};\mu_{t-1}^{\left(1\right)},\sigma_{t-1}^{\left(1\right)}+w_{1}\right),$$

(39) $$p\left(x_{t}^{\left(1\right)}|H_{t}=2,O_{t-1}\right)=N\left(x_{t}^{\left(1\right)};0,w_{2}\right).$$

To estimate the update equations and obtain delta like learning rules equivalent to the ones originally presented in Nassar et al. (2010) we will slightly change the procedure that we followed above for deriving update equations of the HGF. We will first start by computing the update equations for the change point probability Ω_*t*_. Note that *H*_*t*_ is a discrete variable, hence if we integrated out $x_{t}^{\left(1\right)}$ from the posterior distribution in Equations (34) and (35), we would obtain the following relation

(40) $$q\left(H_{t}|\Omega_{t}\right)=p\left(H_{t}|O_{t}\right)=\frac{p\left(o_{t}|H_{t},O_{t-1}\right)p\left(H_{t}\right)}{p\left(o_{t}|O_{t-1}\right)}$$

where observation likelihoods *p*(*o*_*t*_|*H*_*t*_, *O*_*t* − 1_) for *H*_*t*_ = 1, 2 become

(41) $$\;p\left(o_{t}|H_{t}=1,O_{t-1}\right)=N\left(o_{t};\mu_{t-1}^{\left(1\right)},\sigma_{t-1}^{\left(1\right)}+w_{1}+s\right),$$

(42) $$p\left(o_{t}|H_{t}=2,O_{t-1}\right)=N\left(o_{t};0,s+w_{2}\right),$$

Hence we can express the variational probability *q*(*H*_*t*_|Ω_*t*_) as

(43) $$q\left(H_{t}|\Omega_{t}\right)=\frac{{\left[p\left(o_{t}|1,O_{t-1}\right)(1-h)\right]}^{\delta_{1,H_{t}}}{\left[p\left(o_{t}|2,O_{t-1}\right)h\right]}^{\delta_{2,H_{t}}}}{p\left(o_{t}|1,O_{t-1}\right)(1-h)+p\left(o_{t}|2,O_{t-1}\right)h}.$$

By comparing this expression with the assumption about the functional form of the variational distribution shown in Equation (37), we obtain the change point probability as

(44) $$\Omega_{t}=\frac{N\left(o_{t};0,s+w_{2}\right)h}{\begin{array}{l} N\left(o_{t};\mu_{t-1}^{\left(1\right)},\sigma_{t-1}^{\left(1\right)}+w_{1}+s\right)(1-h)\\ \;+\;N\left(o_{t};0,s+w_{2}\right)h \end{array}}.$$

Having obtained one of the factors of the approximate posterior we can apply the same relation between the variational energy and variational distribution as before (see Equation 24), thus

(45) $$\begin{array}{l} q\left(x_{t}^{\left(1\right)}|\lambda_{t}^{\left(1\right)}\right)\propto {\text{e}}^{I\left(x_{t}^{\left(1\right)}\right)},\\ \;I\left(x_{t}^{\left(1\right)}\right)={\langle \ln p\left(o_{t},x_{t}^{\left(1\right)},H_{t}|O_{t-1}\right)\rangle }_{q\left(H_{t}|O_{t-1}\right)},\\ \;=\left(1-\Omega_{t}\right)\ln p\left(o_{t},x_{t}^{\left(1\right)}|H_{t}=1,O_{t-1}\right)\\ \;+\Omega_{t}\ln p\left(o_{t},x_{t}^{\left(1\right)}|H_{t}=2,O_{t-1}\right). \end{array}$$

Using the definition of conditional probability we can express $p\left(o_{t},x_{t}^{\left(1\right)}|H_{t}=1,O_{t-1}\right)$ as

(46) $$\begin{array}{l} p\left(o_{t},x_{t}^{\left(1\right)}|H_{t}=1,O_{t-1}\right)=p\left(x_{t}^{\left(1\right)}|H_{t}=1,O_{t}\right)\\ \;p\left(o_{t}|H_{t}=1,O_{t-1}\right). \end{array}$$

As only the conditional posterior $p\left(x_{t}^{\left(1\right)}|H_{t},O_{t}\right)$ is a function of $x_{t}^{\left(1\right)}$ we obtain the following relation

(47) $$q\left(x_{t}^{\left(1\right)}|\lambda_{t}^{\left(1\right)}\right)\propto p{\left(x_{t}^{\left(1\right)}|H_{t}=1,O_{t}\right)}^{1-\Omega_{t}}p{\left(x_{t}^{\left(1\right)}|H_{t}=2,O_{t}\right)}^{\Omega_{t}}.$$

If we assume for now that the conditional posterior $p\left(x_{t}^{\left(1\right)}|H_{t},O_{t}\right)$ can be expressed as a normal distribution, that is,

(48) $$p\left(x_{t}^{\left(1\right)}|H_{t},O_{t}\right)=N\left(x_{t}^{\left(1\right)};\mu_{t}^{\left(1\right)}|H_{t},\sigma_{t}^{\left(1\right)}|H_{t}\right),$$

we obtain that

(49) $$\begin{array}{l} q\left(x_{t}^{\left(1\right)}|\lambda_{t}^{\left(1\right)}\right)=N(x_{t}^{\left(1\right)};\frac{\frac{1-\Omega_{t}}{\sigma_{t}^{\left(1\right)}|1}\mu_{t}^{\left(1\right)}|1+\frac{\Omega_{t}}{\sigma_{t}^{\left(1\right)}|2}\mu_{t}^{\left(1\right)}|2}{\frac{1-\Omega_{t}}{\sigma_{t}^{\left(1\right)}|1}+\frac{\Omega_{t}}{\sigma_{t}^{\left(1\right)}|2}},\\ \;{\left[\frac{1-\Omega_{t}}{\sigma_{t}^{\left(1\right)}|1}+\frac{\Omega_{t}}{\sigma_{t}^{\left(1\right)}|2}\right]}^{-1}). \end{array}$$

Given that the conditional predictive distributions (Equations 38 and 39) are indeed normal distributions, it is trivial to show that the condition posterior will also be a normal distribution. We obtain the conditional posterior expectations as

(50) $$\mu_{t}^{\left(1\right)}|H_{t}=\{\begin{array}{cc} \mu_{t}^{\left(1\right)}+\frac{\sigma_{t}^{\left(1\right)}|H_{t}}{s}\left(o_{t}-\mu_{t}^{\left(1\right)}\right), & \text{if }H_{t}=1,\\ \frac{\sigma_{t}^{\left(1\right)}|H_{t}}{s}o_{t}, & \text{if }H_{t}=2, \end{array}$$

and conditional posterior uncertainty as

(51) $$\sigma_{t}^{\left(1\right)}|H_{t}=\{\begin{array}{c} \begin{array}{cc} \frac{s\left(\sigma_{t-1}^{\left(1\right)}+w_{1}\right)}{s+w_{1}+\sigma_{t-1}^{\left(1\right)}}, & \text{if }H_{t}=1,\\ \frac{s}{\frac{s}{w_{2}}+1}, & \text{if }H_{t}=2, \end{array} \end{array}$$

where we will assume that *w*_2_ ≫ *s*, hence $\sigma_{t}^{\left(1\right)}|2\approx s$. Substituting Equations (50) and (51) gives us the update equations for the CPM shown in Equation (4). Note that the procedure that we used to derivation of the update equation of the CPM is closely related to the Bayesian forgetting method that is typically applied to the online parameter estimation of the non-stationary processes (Kulhavỳ and Zarrop, 1993; Payzan-LeNestour, 2010).

For the CPM the surprise (negative marginal log-likelihood) of the current observation can be computed exactly as

(52) $$\begin{array}{l} -\ln p\left(o_{t}|O_{t-1},\theta \right)=-\ln[N\left(o_{t};\mu_{t-1}^{\left(1\right)},\sigma_{t-1}^{\left(1\right)}+w_{1}+s\right)\\ \;(1-h)+N\left(o_{t};0,s+w_{2}\right)h], \end{array}$$

where θ denotes the set of fixed parameters of the perceptual model $\left\{\mu_{0}^{\left(1\right)},\sigma_{0}^{\left(1\right)},s,w_{1},w_{2},h\right\}$. As before the optimal parameter values of the perceptual model are obtained by minimizing total surprise over *N* experimental blocks of duration *T* under some prior assumptions *p*(θ) about the parameter values (see Equations 30 and 31).

### 4.2. Model evidence and parameter estimation

To simulate behavioral responses we have used a simple formulation of the response model (see Equation 7) for which the corresponding response likelihood over *T* responses is defined as

(53) $$p\left(R_{T}|O_{T},\theta_{m},\sigma_{r},m\right)=\prod_{t=1}^{T}N\left(r_{t};\mu_{t}^{\left(1\right)}\left(\mu_{t-1}^{\left(1\right)},o_{t},\theta_{m}\right),\sigma_{r}\right),$$

where *R*_*T*_ denotes the set of responses during an experimental block of duration *T*, *O*_*T*_ denotes the set of stimuli (observations) presented to a simulated participant, and θ_*m*_ the set of free parameters of either HGF (*m* = 1) or CPM (*m* = 2).

The Bayesian model comparison is based on computing the model evidence for each model *m* that is defined as the marginal probability of the joint distribution of responses and free model parameters. Thus, model evidence is obtained as

(54) $$p\left(R_{T}|O_{T},m\right)=\int p\left(R_{T}|O_{T},\rho ,m\right)p(\rho )\text{d}\rho ,$$

where we use ρ to denote the full set of free model parameters (the parameters of the perceptual plus the response model), and where *p*(ρ) denotes prior probability. As the integral on the right hand side is invariant under parameter transform we have used the following representation of the free model parameters

(55) $$\rho =\phi (x)=\{\begin{array}{ll} x, & \text{for }x=\mu_{0}^{\left(1\right)},\mu_{0}^{\left(2\right)},\\ \ln x, & \text{for }x=s,w_{1},w_{2},\eta ,\sigma_{0}^{\left(1\right)},\sigma_{0}^{\left(2\right)},\\ \ln\frac{x}{1-x}, & \text{for }x=h. \end{array}$$

This re-parametrization of model parameters allowed us to express both prior and posterior parameter probability as a multivariate normal distribution. For the free model parameters we have set the prior distribution to

(56) $$p(\rho )=\prod_{x}N\left(\phi (x);\mu (x),\sigma (x)\right),$$

where

$$\mu (x)=\{\begin{array}{l} \begin{array}{ll} 0 & \text{for }x=\mu_{0}^{\left(1\right)},\mu_{0}^{\left(2\right)},s,w_{1},\sigma_{0}^{\left(1\right)},\sigma_{0}^{\left(2\right)}\\ -2 & \text{for }x=\eta \\ 7 & \text{for }x=w_{2}\\ -3 & \text{for }x=h \end{array} \end{array}$$

and

$$\sigma (x)=\{\begin{array}{l} \begin{array}{ll} 5 & \text{for}x=\mu_{0}^{\left(1\right)},\mu_{0}^{\left(2\right)},s,w_{1},w_{2},\eta ,\sigma_{0}^{\left(1\right)},\sigma_{0}^{\left(2\right)}\\ 2 & \text{for}x=h \end{array} \end{array}$$

The reason for using different prior assumption for *w*_2_, *h*, and η is that we had specific prior assumptions about these parameters, specifically we expected that *w*_2_ ≫ *s*, that *h* < 0.5, and that η < 1. Nevertheless, this allowed for modest improvement of the inference accuracy of these three parameter values.

In the case of the Bayesian estimation of model evidence we will apply the Laplace approximation to the integral on the right hand side of Equation (54). Hence, we approximately estimate the model log-evidence as

(57) $$\ln p\left(R_{T}|O_{T},m\right)\approx \ln p\left(R_{T},\rho^{*}|O_{T},m\right)+\frac{1}{2}\ln|2\pi S\left(\rho^{*}\right)|,$$

where

(58) $$\begin{array}{l} \rho^{*}=\arg\underset{\rho }{\max}\ln p\left(R_{T}|\rho ,O_{T},m\right);\\ S^{-1}\left(\rho \right)=-\frac{\partial^{2}\ln p\left(R_{T},\rho |O_{T},m\right)}{\partial \rho^{2}}. \end{array}$$

Under the Laplace approximation the posterior probability of model parameters is then expressed as the multivariate normal distribution centered at ρ^*^ and with covariance *S*(ρ^*^), that is,

(59) $$p\left(\rho |R_{T},O_{T},m\right)=N\left(\rho ;\rho^{*},S(\rho^{*})\right).$$

For the maximum likelihood parameter estimation we have considered the BIC as an approximation to the model evidence [the BIC is actually derived from the Laplace approximation itself (Wit et al., 2012)]. Hence, in this second variant we obtain the model evidence as

(60) $$\ln p\left(R_{T}|O_{T},m\right)\approx \ln p\left(R_{T}|\rho^{*},O_{T},m\right)-\frac{d}{2}\ln T,$$

where *d* denotes the number of free parameters, and ρ^*^ denotes the maximum of the log-likelihood, that is

(61) $$\rho^{*}=\arg\underset{\rho }{\max}\ln p\left(R_{T}|\rho ,O_{T},m\right).$$

Similar as for the Laplace approximation the uncertainty of the parameter estimate, denoted with ρ^*^, is obtained as

(62) $$\sigma_{\rho }^{-1}=-\frac{\partial^{2}\ln p\left(R_{T}|\rho ,O_{T},m\right)}{\partial \rho^{2}}{|}_{\rho =\rho^{*}}.$$

Finding the maxima of either log-likelihood or the joint probability distribution is analytically intractable, as both functions normally have multiple modes. Hence, we have performed only numerical estimation both of the position of the maxima and of the second derivatives around the maxima. For numerical optimization of objective functions (log-likelihood or log-joint probability) we have used an open-source implementation of the CMA-ES algorithm (Hansen, 2006, 2015), whereas for the numerical estimation of the Hessian (second derivative around the mode) we have used an open-source library for numerical differentiation (Brodtkorb and D'Errico, 2015).

## Author contributions

Concept and design of the study: DM and SK. Implementation, data acquisition, and analysis: DM. Data interpretation and writing of the paper: DM and SK.

## Funding

This work was supported by the US-German Collaboration in Computational Neuroscience of NSF and BMBF (Förderkennzeichen: 01GQ1205, to SJK), open access publication fund of the TU Dresden, and the German Research Foundation (DFG).

### Conflict of interest statement

The authors declare that the research was conducted in the absence of any commercial or financial relationships that could be construed as a potential conflict of interest.

## References

[B1] Acerbi L. Vijayakumar S. Wolpert D. M. (2014). On the origins of suboptimality in human probabilistic inference. PLoS Comput. Biol. 10:e1003661. 10.1371/journal.pcbi.1003661 24945142 PMC4063671

[B2] Adams R. P. MacKay D. J. (2007). Bayesian Online Changepoint Detection. Cambridge, UK: University of Cambridge.

[B3] Angela J. Y. (2007). Adaptive behavior: humans act as bayesian learners. Curr. Biol. 17, R977–R980. 10.1016/j.cub.2007.09.007 18029257

[B4] Behrens T. E. J. Woolrich M. W. Walton M. E. Rushworth M. F. S. (2007). Learning the value of information in an uncertain world. Nat. Neurosci. 10, 1214–1221. 10.1038/nn1954 17676057

[B5] Bland A. R. Schaefer A. (2012). Different varieties of uncertainty in human decision-making. Front. Neurosci. 6:85. 10.3389/fnins.2012.00085 22701401 PMC3370661

[B6] Box G. E. P. Tiao G. C. (1992). Bayesian Inference in Statistical Analysis, Vol. 40. New York, NY: John Wiley & Sons.

[B7] Brodtkorb P. A. D'Errico J. (2015). numdifftools 0.9.12. Available online at: https://github.com/pbrod/numdifftools

[B8] Carlin B. P. Louis T. A. (1997). Bayes and empirical bayes methods for data analysis. Stat. Comput. 7, 153–154. 10.1023/A:1018577817064

[B9] Chickering D. M. Heckerman D. (1997). Efficient approximations for the marginal likelihood of bayesian networks with hidden variables. Mach. Learn. 29, 181–212. 10.1023/A:1007469629108

[B10] Daunizeau J. Den Ouden H. E. M. Pessiglione M. Kiebel S. J. Friston K. J. Stephan K. E. (2010a). Observing the observer (II): deciding when to decide. PLoS ONE 5:e15555. 10.1371/journal.pone.0015555 21179484 PMC3001882

[B11] Daunizeau J. Den Ouden H. E. M. Pessiglione M. Kiebel S. J. Stephan K. E. Friston K. J. (2010b). Observing the observer (I): Meta-Bayesian models of learning and decision-making. PLoS ONE 5:e15554. 10.1371/journal.pone.0015554 21179480 PMC3001878

[B12] Daunizeau J. Preuschoff K. Friston K. J. Stephan K. E. (2011). Optimizing experimental design for comparing models of brain function. PLoS Comput. Biol. 7:e1002280. 10.1371/journal.pcbi.1002280 22125485 PMC3219623

[B13] Diaconescu A. O. Mathys C. Weber L. A. E. Daunizeau J. Kasper L. Lomakina E. I.. (2014). Inferring on the intentions of others by hierarchical bayesian learning. PLoS Comput. Biol. 10:e1003810. 10.1371/journal.pcbi.1003810 25187943 PMC4154656

[B14] Doya K. (2008). Modulators of decision making. Nat. Neurosci. 11, 410–416. 10.1038/nn2077 18368048

[B15] Friston K. Mattout J. Trujillo-Barreto N. Ashburner J. Penny W. (2007). Variational free energy and the laplace approximation. Neuroimage 34, 220–234. 10.1016/j.neuroimage.2006.08.035 17055746

[B16] Friston K. J. Penny W. (2003). Posterior probability maps and SPMs. Neuroimage 19, 1240–1249. 10.1016/S1053-8119(03)00144-7 12880849

[B17] Glaze C. M. Kable J. W. Gold J. I. (2015). Normative evidence accumulation in unpredictable environments. eLife 4:e08825. 10.7554/eLife.08825 26322383 PMC4584511

[B18] Hansen N. (2006). The CMA evolution strategy: a comparing review, in Towards a New Evolutionary Computation, eds Lozano J. A. Larra1aga P. Inza I. Bengoetxea E. (Berlin; Heidelberg: Springer), 75–102.

[B19] Hansen N. (2015). cma 1.1.06. Available online at: https://pypi.python.org/pypi/cma

[B20] Horbelt W. (2001). Maximum Likelihood Estimation in Dynamical Systems. Ph.D thesis, University of Freiburg.

[B21] Iglesias S. Mathys C. Brodersen K. H. Kasper L. Piccirelli M. den Ouden H. E. M.. (2013). Hierarchical prediction errors in midbrain and basal forebrain during sensory learning. Neuron 80, 519–530. 10.1016/j.neuron.2013.09.009 24139048

[B22] Judd K. (2007). Failure of maximum likelihood methods for chaotic dynamical systems. Phys. Rev. E Stat. Nonlin. Soft. Matter Phys. 75:036210. 10.1103/PhysRevE.75.036210 17500772

[B23] Körding K. P. Wolpert D. M. (2004). The loss function of sensorimotor learning. Proc. Natl. Acad. Sci. U.S.A. 101, 9839–9842. 10.1073/pnas.0308394101 15210973 PMC470761

[B24] Kulhavỳ R. Zarrop M. B. (1993). On a general concept of forgetting. Int. J. Control 58, 905–924. 10.1080/00207179308923034

[B25] Lewi J. Butera R. Paninski L. (2009). Sequential optimal design of neurophysiology experiments. Neural Comput. 21, 619–687. 10.1162/neco.2008.08-07-594 18928364

[B26] Lomakina E. I. Paliwal S. Diaconescu A. O. Brodersen K. H. Aponte E. A. Buhmann J. M.. (2015). Inversion of hierarchical Bayesian models using Gaussian processes. Neuroimage 118, 133–145. 10.1016/j.neuroimage.2015.05.084 26048619

[B27] Mathys C. Daunizeau J. Friston K. J. Stephan K. E. (2011). A bayesian foundation for individual learning under uncertainty. Front. Hum. Neurosci. 5:39. 10.3389/fnhum.2011.00039 21629826 PMC3096853

[B28] Mathys C. D. Lomakina E. I. Daunizeau J. Iglesias S. Brodersen K. H. Friston K. J.. (2014). Uncertainty in perception and the hierarchical gaussian filter. Front. Hum. Neurosci. 8:825. 10.3389/fnhum.2014.00825 25477800 PMC4237059

[B29] McGuire J. T. Nassar M. R. Gold J. I. Kable J. W. (2014). Functionally dissociable influences on learning rate in a dynamic environment. Neuron 84, 870–881. 10.1016/j.neuron.2014.10.013 25459409 PMC4437663

[B30] Nassar M. R. Gold J. I. (2013). A healthy fear of the unknown: perspectives on the interpretation of parameter fits from computational models in neuroscience. PLoS Comput. Biol. 9:e1003015. 10.1371/journal.pcbi.1003015 23592963 PMC3617224

[B31] Nassar M. R. Rumsey K. M. Wilson R. C. Parikh K. Heasly B. Gold J. I. (2012). Rational regulation of learning dynamics by pupil-linked arousal systems. Nat. Neurosci. 15, 1040–1046. 10.1038/nn.3130 22660479 PMC3386464

[B32] Nassar M. R. Wilson R. C. Heasly B. Gold J. I. (2010). An approximately Bayesian delta-rule model explains the dynamics of belief updating in a changing environment. J. Neurosci. 30, 12366–12378. 10.1523/JNEUROSCI.0822-10.2010 20844132 PMC2945906

[B33] O'Doherty J. P. Hampton A. Kim H. (2007). Model-based fmri and its application to reward learning and decision making. Ann. N.Y. Acad. Sci. 1104, 35–53. 10.1196/annals.1390.022 17416921

[B34] Paliwal S. Petzschner F. H. Schmitz A. K. Tittgemeyer M. Stephan K. E. (2014). A model-based analysis of impulsivity using a slot-machine gambling paradigm. Front. Hum. Neurosci. 8:428. 10.3389/fnhum.2014.00428 25071497 PMC4080386

[B35] Payzan-LeNestour E. (2010). Bayesian learning in unstable settings: experimental evidence based on the bandit problem. Swiss Finance Ins. Res. Paper 10, 1–41. Available online at: http://EconPapers.repec.org/RePEc:chf:rpseri:rp1028

[B36] Payzan-LeNestour E. Bossaerts P. (2011). Risk, unexpected uncertainty, and estimation uncertainty: Bayesian learning in unstable settings. PLoS Comput. Biol. 7:e1001048. 10.1371/journal.pcbi.1001048 21283774 PMC3024253

[B37] Payzan-LeNestour E. Dunne S. Bossaerts P. O'Doherty J. P. (2013). The neural representation of unexpected uncertainty during value-based decision making. Neuron 79, 191–201. 10.1016/j.neuron.2013.04.037 23849203 PMC4885745

[B38] Penny W. (2012). Comparing dynamic causal models using AIC, BIC and free energy. Neuroimage 59, 319–330. 10.1016/j.neuroimage.2011.07.039 21864690 PMC3200437

[B39] Qian T. Aslin R. N. (2014). Learning bundles of stimuli renders stimulus order as a cue, not a confound. Proc. Natl. Acad. Sci. U.S.A. 111, 14400–14405. 10.1073/pnas.1416109111 25246587 PMC4210001

[B40] Quinn A. Karny M. (2007). Learning for non-stationary dirichlet processes. Int. J. Adap. Control Signal Proces. 21, 827. 10.1002/acs.949

[B41] Rigoux L. Stephan K. E. Friston K. J. Daunizeau J. (2014). Bayesian model selection for group studies - revisited. Neuroimage 84, 971–985. 10.1016/j.neuroimage.2013.08.065 24018303

[B42] Rosa M. J. Bestmann S. Harrison L. Penny W. (2010). Bayesian model selection maps for group studies. Neuroimage 49, 217–224. 10.1016/j.neuroimage.2009.08.051 19732837 PMC2791519

[B43] Stephan K. E. Penny W. D. Daunizeau J. Moran R. J. Friston K. J. (2009). Bayesian model selection for group studies. Neuroimage 46, 1004–1017. 10.1016/j.neuroimage.2009.03.025 19306932 PMC2703732

[B44] Summerfield C. Behrens T. E. Koechlin E. (2011). Perceptual classification in a rapidly changing environment. Neuron 71, 725–736. 10.1016/j.neuron.2011.06.022 21867887 PMC3975575

[B45] Vossel S. Mathys C. Daunizeau J. Bauer M. Driver J. Friston K. J.. (2014). Spatial attention, precision, and Bayesian inference: a study of saccadic response speed. Cereb. Cortex 24, 1436–1450. 10.1093/cercor/bhs418 23322402 PMC4014178

[B46] Vossel S. Mathys C. Stephan K. E. Friston K. J. (2015). Cortical coupling reflects bayesian belief updating in the deployment of spatial attention. J. Neurosci. 35, 11532–11542. 10.1523/JNEUROSCI.1382-15.2015 26290231 PMC4540794

[B47] Wilson R. C. Nassar M. R. Gold J. I. (2010). Bayesian online learning of the hazard rate in change-point problems. Neural Comput. 22, 2452–2476. 10.1162/NECO_a_00007 20569174 PMC2966286

[B48] Wilson R. C. Nassar M. R. Gold J. I. (2013). A mixture of delta-rules approximation to bayesian inference in change-point problems. PLoS Comput. Biol. 9:e1003150. 10.1371/journal.pcbi.1003150 23935472 PMC3723502

[B49] Wilson R. C. Niv Y. (2011). Inferring relevance in a changing world. Front. Hum. Neurosci. 5:189. 10.3389/fnhum.2011.00189 22291631 PMC3264902

[B50] Wit E. Heuvel E. V. D. Romeijn J.-W. (2012). ‘All models are wrong…’: an introduction to model uncertainty. Stat. Neerlandica 66, 217–236. 10.1111/j.1467-9574.2012.00530.x

[B51] Woolrich M. W. Jbabdi S. Patenaude B. Chappell M. Makni S. Behrens T.. (2009). Bayesian analysis of neuroimaging data in fsl. Neuroimage 45, S173–S186. 10.1016/j.neuroimage.2008.10.055 19059349

[B52] Yu A. J. Dayan P. (2005). Uncertainty, neuromodulation, and attention. Neuron 46, 681–692. 10.1016/j.neuron.2005.04.026 15944135

