# Supplementary material for: Comparative Analysis of Behavioral Models for Adaptive Learning in Changing Environments
Source: Front Comput Neurosci. 2016 Apr 20;10:33. doi: 10.3389/fncom.2016.00033 (PMC4837154; doi:10.3389/fncom.2016.00033)
Supplement: Supplementary file 1 [file DataSheet1.PDF]

Supplementary Material: Comparative analysis of behavioural models for adaptive learning in changing environments

Dimitrije Marković, and Stefan J. Kiebel

*Correspondence:
Dimitrije Marković
dimitrije.markovic@tu-dresden.de

1 SUPPLEMENTARY FIGURES

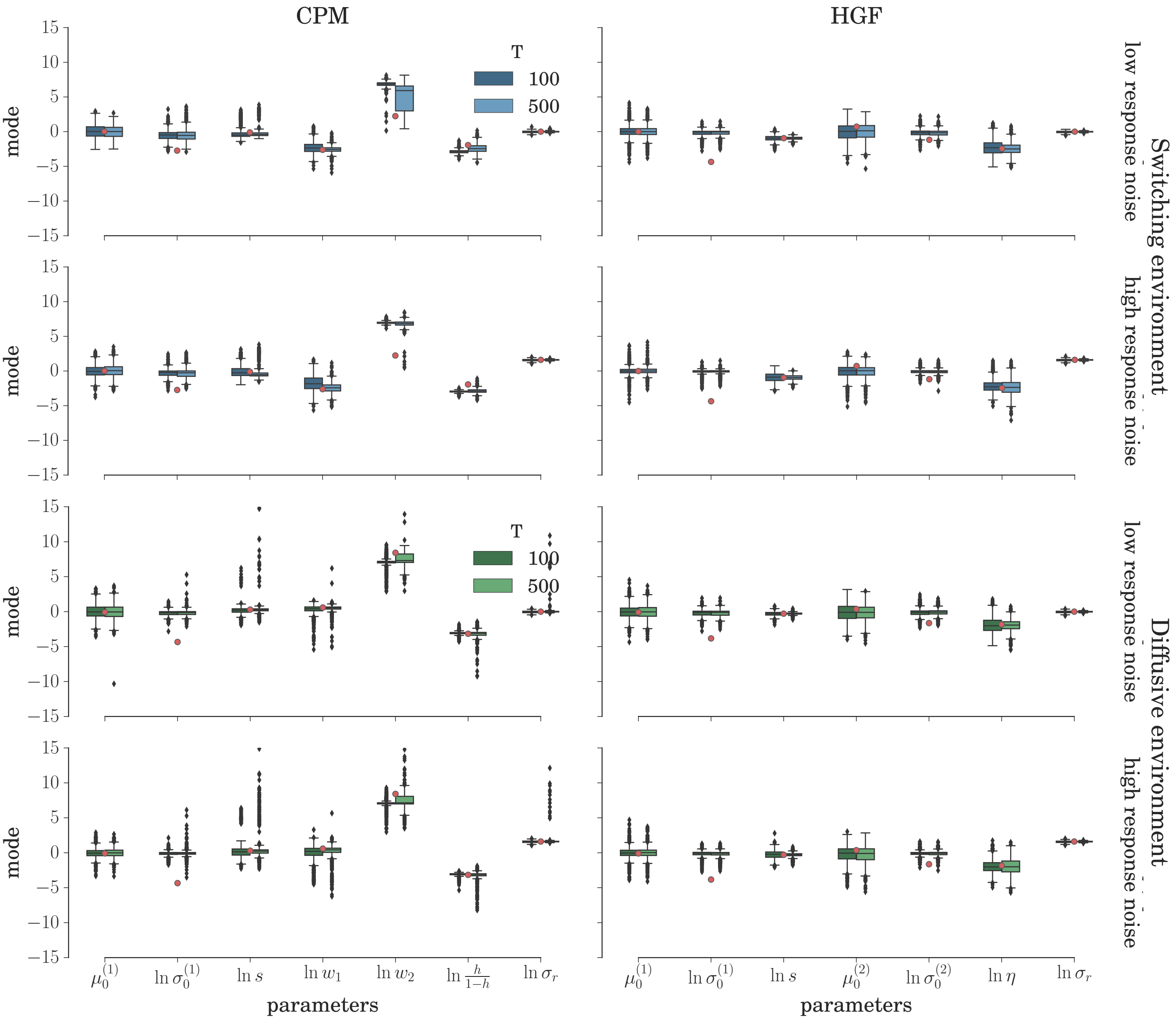

Figure S1. Parameter inference with the BI-LA method: Boxplot of the distribution of the mode of the posterior free parameter probability estimated over $n = 1000$ synthetic agents in each condition (environment, duration, response noise level). The red circles indicate the true parameter value. The boxes span the range from the 25th to the 75th percentile, black horizontal line within each box shows the median, and whiskers span the range from 1.5 of the inter-quartile (IQR) range below the low quartile to the 1.5 IQR above the upper quartile. Diamonds indicate the outliers.

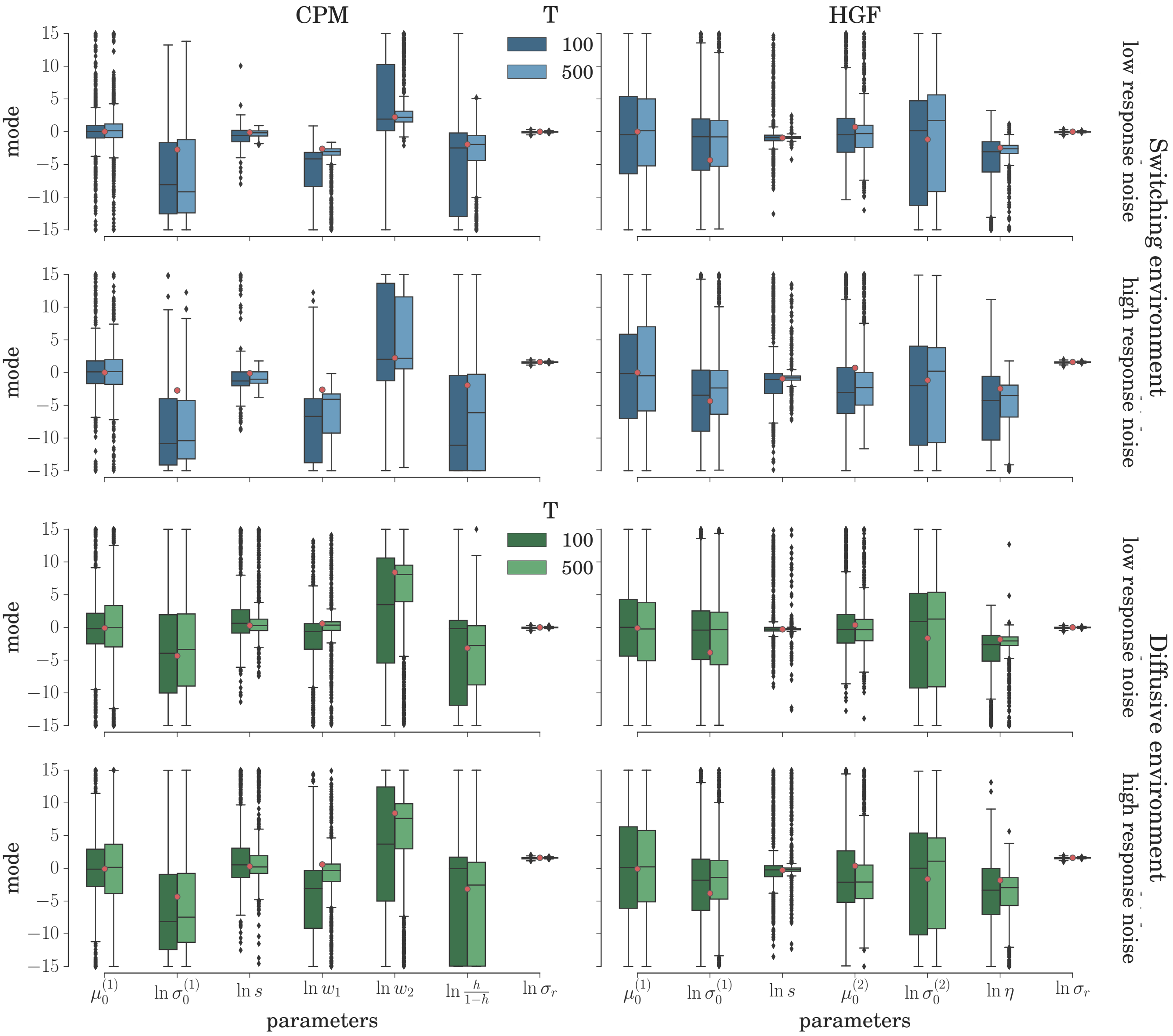

Figure S2. Parameter estimation with the MLE method: Boxplot of the distribution of maximum likelihood value estimated over $n = 1000$ synthetic agents in each condition (environment, duration, response noise level). The red circles indicate the true parameter value. The boxes span the range from the 25th to the 75th percentile, black horizontal line within each box shows the median, and whiskers span the range from 1.5 of the inter-quartile (IQR) range below the low quartile to the 1.5 IQR above the upper quartile. Diamonds indicate the outliers.

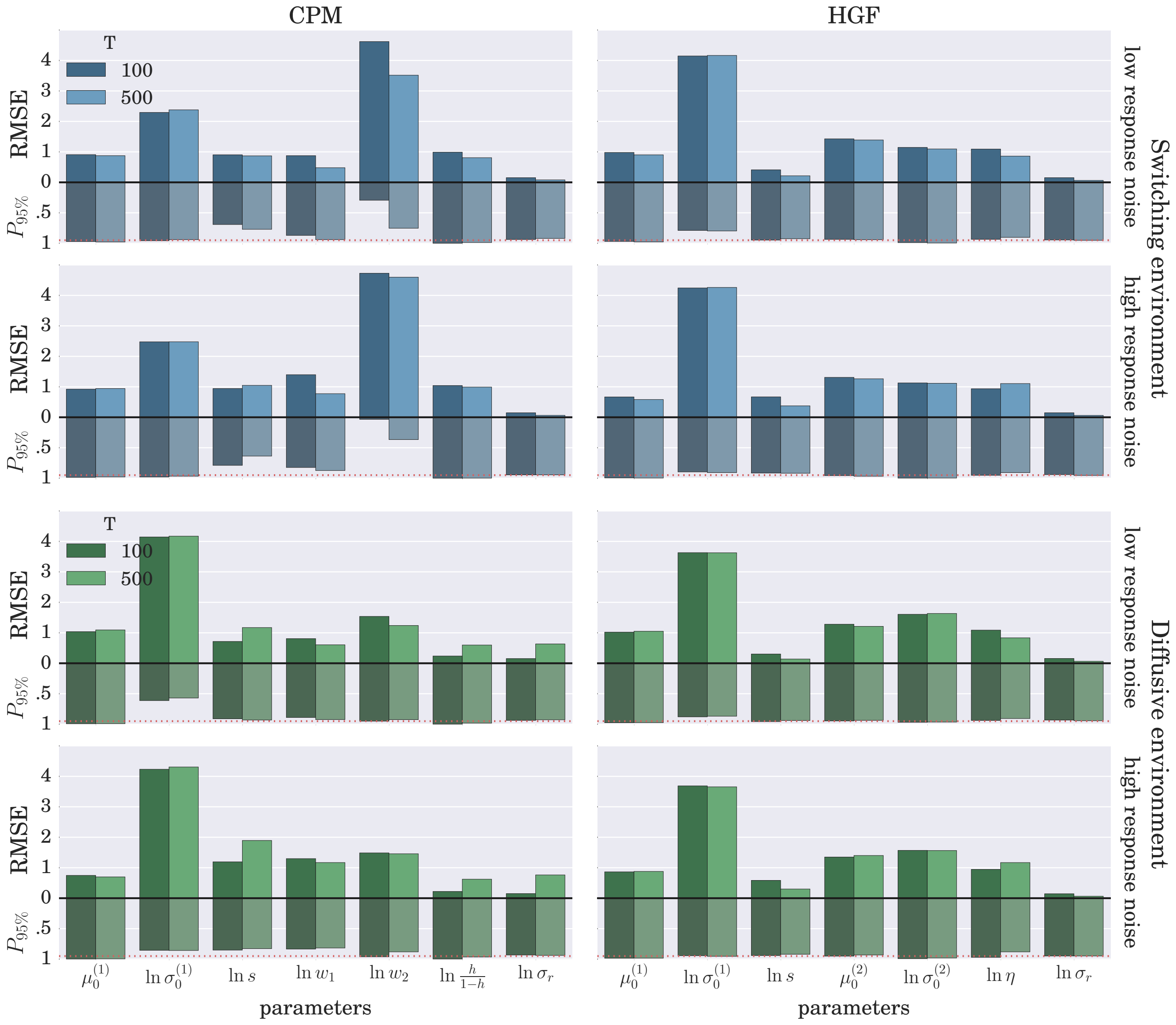

Figure S3. Estimation accuracy for the BI-LA method: Colored bars above the zero line (black solid line) show the root-mean-square error (RMSE) of the posterior mode estimated from the behavioral responses of $n = 1000$ agents in each condition (environment, duration, response noise level). The bars bellow the zero line denote the probability $P_{95\%}$ that the true parameter value is within two standard deviations from the posterior mode. The red dotted line marks the 0.95 probability level.

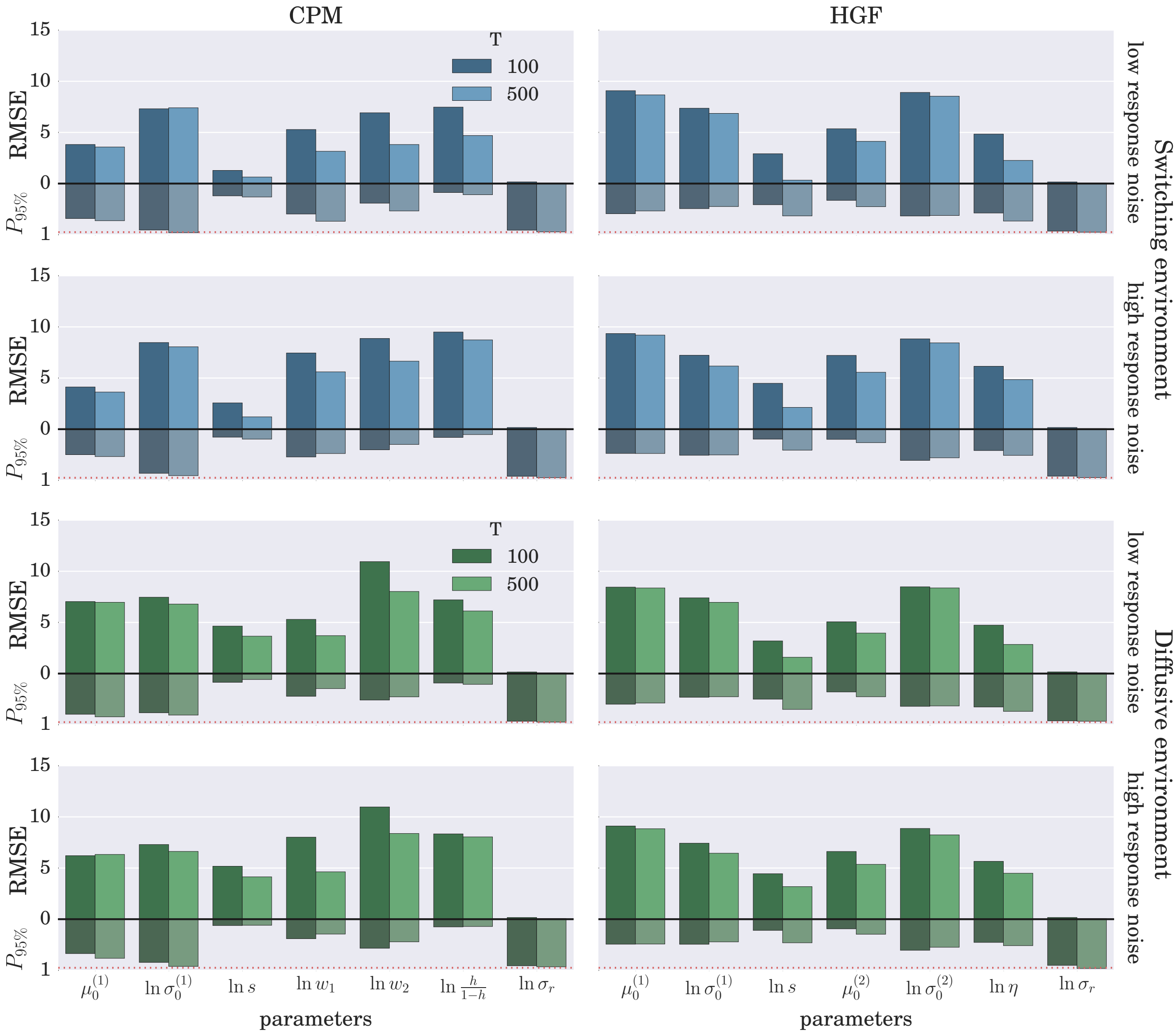

Figure S4. Estimation accuracy for the MLE method: Colored bars above the zero line (black solid line) show the root-mean-square error (RMSE) of the posterior mode estimated from the behavioral responses of $n = 1000$ agents in each condition (environment, duration, response noise level). The bars bellow the zero line denote the probability $P_{95\%}$ that the true parameter value is within two standard deviations from the posterior mode. The red dotted line marks the 0.95 probability level.

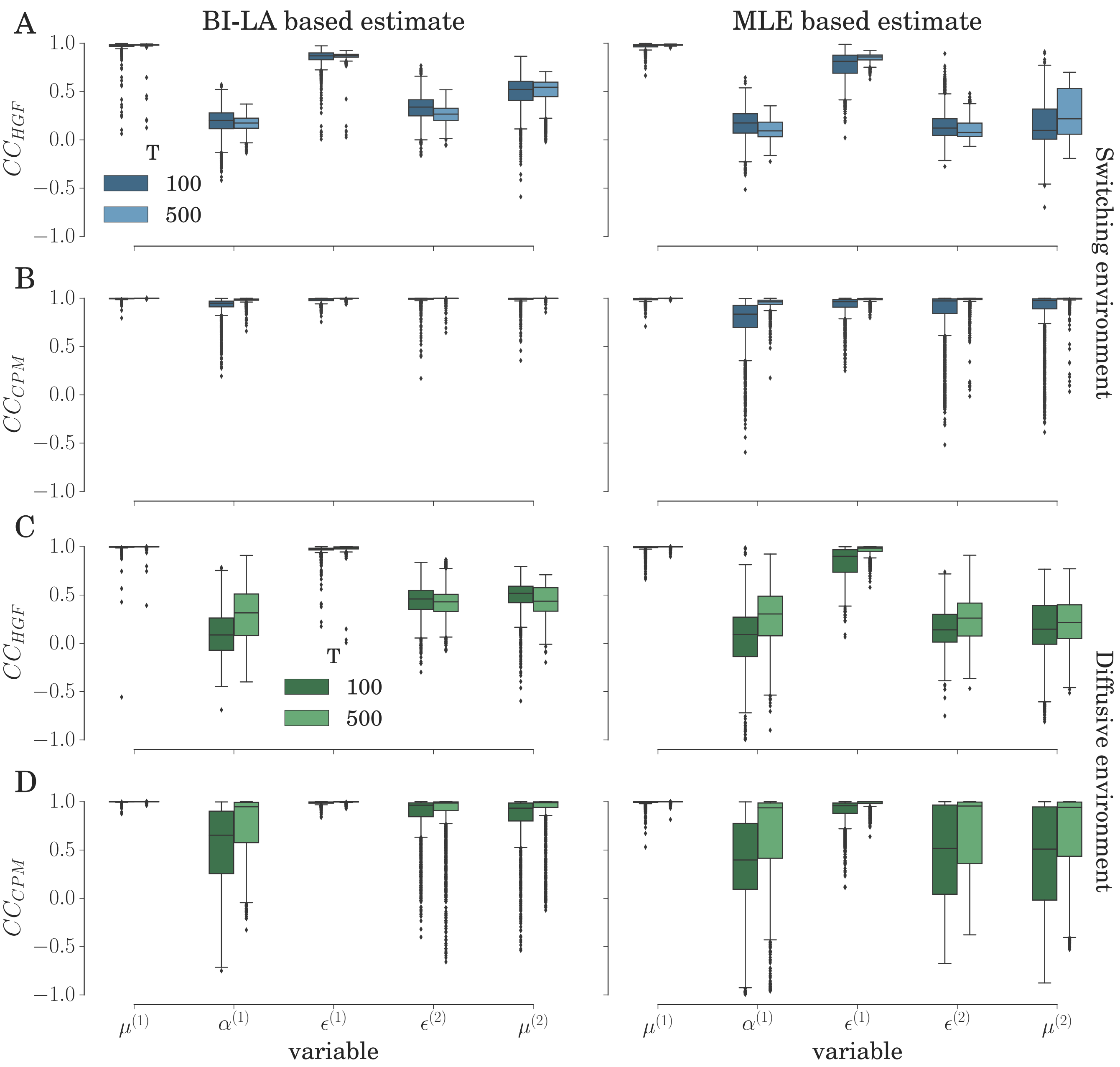

Figure S5. Correlation analysis in the switching (A-B) and diffusive (C-D) experimental environment for the behavior simulated using the CPM model: Distribution of the correlation coefficient between simulated and inferred trajectories of perceptual variables. (A, C) Correlation coefficient (CC_{HGF}) for the cases when behavior was inferred using the HGF and generated using the CPM; (B, D) Correlation coefficient (CC_{CPM}) for the cases when behavior was inferred using the CPM and generated using the CPM. The presented distribution is a combined estimate over the conditions with the low and the high response noise.

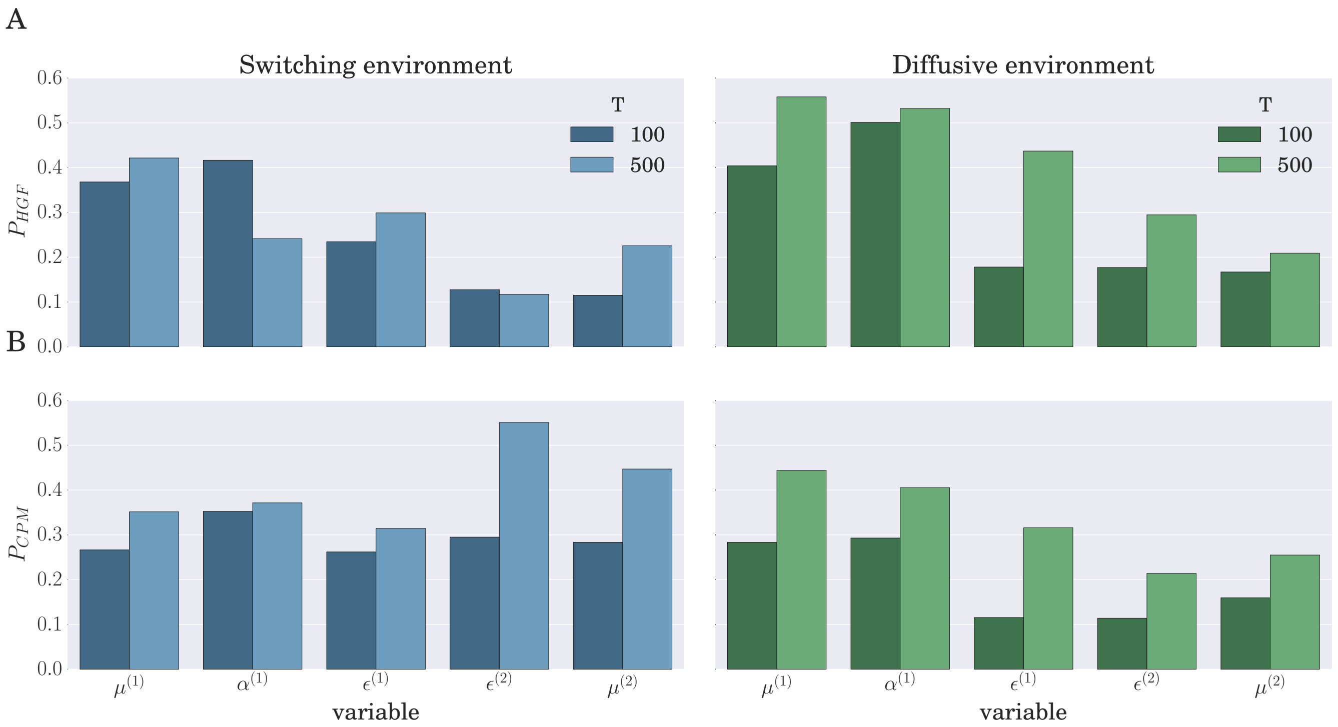

Figure S6. Probability that the MLE based method provides higher correlation then the median correlation of the BI-LA based method in the two experimental environments for the behavior simulated using the CPM model: (A) Exceedance probability P_{HGF} of the MLE based estimate when behavior was inferred using the HGF and generated using the CPM; (B) Exceedance probability P_{CPM} of the MLE based estimate when behavior was inferred using the CPM and generated using the CPM; Values bellow a probability of 0.5 indicate that the BI-LA based median correlation is higher then the MLE based median correlation.
